# Sympathetic-Mediated Intestinal Cell Death Contributes to Gut Barrier Impairment After Stroke

**DOI:** 10.1007/s12975-023-01211-y

**Published:** 2023-11-30

**Authors:** Kathryn Prame Kumar, Liam D. McKay, Huynh Nguyen, Jasveena Kaur, Jenny L. Wilson, Althea R. Suthya, Sonja J. McKeown, Helen E. Abud, Connie H. Y. Wong

**Affiliations:** 1 Centre for Inflammatory Diseases, Department of Medicine, School of Clinical Sciences at Monash Health, Monash Medical Centre, Monash University, Clayton, VIC 3168 Australia; 2 Department of Anatomy and Developmental Biology, Development and Stem Cells Program, Biomedical Discovery Institute, Monash University, Clayton, VIC 3800 Australia

**Keywords:** Cerebral ischaemia, Gut permeability, Brain-gut axis, Epithelium, Barrier integrity

## Abstract

Tissue injury induced by stroke is traditionally thought to be localised to the brain. However, there is an accumulating body of evidence to demonstrate that stroke promotes pathophysiological consequences in peripheral tissues including the gastrointestinal system. In this study, we investigated the mechanisms underlying gut permeability after stroke. We utilised the clinically relevant experimental model of stroke called permanent intraluminal middle cerebral artery occlusion (pMCAO) to examine the effect of cerebral ischaemia on the gut. We detected stroke-induced gut permeability at 5 h after pMCAO. At this timepoint, we observed significantly elevated intestinal epithelial cell death in post-stroke mice compared to their sham-operated counterparts. At 24 h after stroke onset when the gut barrier integrity is restored, our findings indicated that post-stroke intestinal epithelium had higher expression of genes associated with fructose metabolism, and hyperplasia of intestinal crypts and goblet cells, conceivably as a host compensatory mechanism to adapt to the impaired gut barrier. Furthermore, we discovered that stroke-induced gut permeability was mediated by the activation of the sympathetic nervous system as pharmacological denervation decreased the stroke-induced intestinal epithelial cell death, goblet cell and crypt hyperplasia, and gut permeability to baseline levels. Our study identifies a previously unknown mechanism in the brain-gut axis by which stroke triggers intestinal cell death and gut permeability.

**Supplementary Information:**

The online version contains supplementary material available at 10.1007/s12975-023-01211-y.

## Introduction

Stroke is a common and debilitating cerebrovascular event that, in approximately 80% of cases, is caused by the sudden obstruction of blood flow (or ischaemia) to a focal region of the brain. Ischaemic stroke accounts for approximately 10% of all deaths worldwide, and it is the leading cause of disability in Australia [1]. In recent years, an increasing number of studies have revealed the devastating effects of stroke beyond the brain, inducing far-reaching pathophysiological consequences in peripheral tissues including immune, gastrointestinal, respiratory, and endocrine systems [2]. In fact, over half of all patients admitted to the hospital with ischaemic stroke suffer gastrointestinal complications [3–5]. Multiple reports have demonstrated elevated gut vascular and barrier permeability within hours of experimental stroke onset, leading to disruption of intestinal homeostasis and the entry of intestinal luminal content into host tissues [6–9]. This stroke-induced gut permeability is evidenced to promote gut-derived bacterial translocation to other peripheral tissues, including the lung, to cause infection [6]. Whilst this discovery was made in well-defined mouse models of experimental stroke, there is evidence of common commensal gut bacterial detection in the blood or sputum of stroke patients, strongly indicating that impairment of gut barrier integrity contributes to dissemination of gut-derived bacteria to the periphery and development of stroke-associated pneumonia [6]. Despite this, the mechanism by which stroke-induced brain injury alters the intestinal environment distally to compromise gut barrier integrity remains elusive.

The gut barrier is made up of millions of epithelial cells held together by tight junction, adherens junction, and gap junction proteins, and this gut barrier functionally remains selectively permeable to nutrients yet vitally shields the host from constant threats, such as extreme pH conditions, pathobionts in the intestinal lumens and potential inflammatory insults from food products [10–12]. Unlike the thicker epithelial layers in other areas of the body, the single monolayer of cells constituting the intestinal epithelial barrier leaves the host tissue particularly vulnerable to perturbation. To account for this, the intestinal barrier is reliant upon its prolific cell regeneration to replace senescent or dying epithelial cells [13, 14]. Epithelial cell regeneration is mediated by the continuous proliferation of stem cells localised in the invaginations of the intestinal wall termed intestinal crypts. The newly generated intestinal epithelial cells compete to remain within the stem cell zone or migrate upwards and differentiate into mature epithelial cells within the overlying villus. Subsequently, the regional downregulation of adhesion molecules within the apical villus results in the shedding of senescent or dying epithelial cells into the intestinal lumen to undergo a form of contact-mediated apoptosis termed anoikis [15]. Therefore, epithelial cell regeneration and migration in the intestinal crypts and villi are central to the self-renewing and protective functionality of the intestinal barrier [13, 14]. Indeed, dysregulation of epithelial cell metabolism, proliferation, or differentiation can trigger gut barrier discontinuity, which may lead to more paracellular routes for bacterial translocation into the host tissue [16].

The autonomic nervous system, bacterial activity of the microbiota, and mucosal inflammation are important factors that regulate epithelial proliferation [17–19]. In the settings of traumatic brain injury, there is clinical evidence of microbial dysbiosis, intestinal inflammation, and sympathetic hyperactivation, and such events could explain the occurrence of excessive epithelial shedding in the intestines of rats [20, 21]. Similarly, these hallmarks of metabolic, microbial, and immune and nervous system dysfunction were observed in mice and patients after stroke [22–25]. However, little is known about how these systemic effects of stroke impact intestinal epithelial cell function. In this study, we hypothesised that the stroke-induced activation of the sympathetic nervous system (SNS) and microbial dysbiosis disrupts intestinal epithelial cell metabolism, proliferation, and turnover, consequentially generating a microenvironment prone to impaired gut barrier integrity.

## Material and Methods

The data that support the findings of this study are available from the corresponding author upon reasonable request. All experiments were performed in compliance with the institutional guidelines and approved by Monash University Animal Ethics Committee. We randomly allocated animals to various groups by the coin-flip approach. The animal allocation was concealed to the investigators who performed data analysis. All authors had access to the study data and had reviewed and approved the final manuscript. Additional methods information can be found in Supplementary Information.

### Animals

Specific-pathogen free (SFP) C57BL/6J and *Adrb2*^*-/-*^ mice at 8–12 weeks old were obtained from Monash Animal Research Platform (MARP) or bred in-house and housed in a SPF facility at Monash Medical Centre Animal Facility (MMCAF; Clayton, VIC, Australia). Additionally, we obtained germ-free (GF) C57BL/6J mice from Germ Free Unit of Walter and Eliza Hall Institute of Medical Research (WEHI; Kew, VIC, Australia). *Adrb2*^*-/-*^ mice were initially obtained as *Adrb1/2*^*-/-*^ double knockout on a mixed background from Jackson Laboratory (RRID:IMSR_JAX:003810). We subsequently bred *Adrb2*^*-/-*^ single knockout mice and backcrossed them with C57BL/6J mice for ten generations. All animals used were male unless stated otherwise. Animals were housed under a 12-h light-dark cycle in a temperature-controlled environment with access to food and water *ad libitum*. All procedures described here were approved by the Monash University Animal Ethics Committee (MMCB/2016/10, MMCB/2018/002 and MMCB/2018/25BC).

### Middle Cerebral Artery Occlusion (MCAO) Model

To model cerebral ischaemia in rodents, mice underwent the permanent middle cerebral artery occlusion (pMCAO) surgery [6]. Mice were anaesthetised with intraperitoneal administration of 150 mg/kg ketamine and 10 mg/kg xylazine, and placed on a thermostatically regulated heat pad in order to maintain its body temperature at 37°C throughout the procedure. Hair was removed at the neck and the incision site was sterilised using 80% ethanol. A 10-mm incision was made at the neck and the right common carotid artery, external carotid artery, and internal carotid artery were isolated from the connective tissue. Blood flow to the common carotid artery and internal carotid artery was temporarily occluded using a vessel clip and a silk suture tension line, respectively. A silicon-coated monofilament (Doccol Corporation) with a diameter of 0.21–0.23 mm was threaded into the external carotid artery to occlude the blood flow of MCA. More than 70% drop in cerebral blood flow was confirmed by laser Doppler flowmetry. The neck incision was closed with sutures and the animal was left to recover from anaesthesia on a heating pad maintained at 37°C. As a surgical control, sham-operated animals underwent the same procedure except arterial occlusion. All animals had access to dishes of mash and water *ad libitum*, and were monitored closely after surgery. Animals that died unexpectedly during or after surgery were excluded from the study. To examine the contribution of the sympathetic nervous system on gut dysfunction post-stroke, 6-hydroxydopamine (6-OHDA; Sigma) diluted in saline (vehicle control) was injected intraperitoneally at a concentration of 100 mg/kg 3 days prior to sham or pMCAO surgery to chemically sympathectomise peripheral nerve terminals containing catecholamines.

### Gut Permeability Assay

To examine the extent of gut permeability after cerebral ischaemia, mice were orally gavaged with 500 mg/kg of the 4.4-kDa fluorescein-isothiocyanate-labelled dextran (FITC-dextran, Sigma) at 1 h after surgery. Four hours later, mice were anaesthetised with isoflurane, and blood was collected via cardiac puncture and deposited into an Eppendorf tube which was then inverted briefly and left to clot at room temperature (RT) for 5 min. Blood samples were centrifuged at 2.4*g* for 5 min at RT, and the serum phase was then collected and stored at −80°C until use. Sera were read at a gain of 750, excitation of 485, and emission of 520 nm on an Infinite M1000 PRO microplate reader (Tecan). A standard curve of known FITC-dextran concentrations with associated fluorescent absorbance was used to determine the FITC-dextran concentration of the samples. Each data point represents the average of two technical replicates of FITC–dextran concentration of each animal.

### Intravital Microscopy of the Ileum to Assess Blood Flow

To assess if stroke-induced gut permeability is due to changes in intestinal blood flow, we performed intravital microscopy of the ileum as previously described [26] and also revised. Briefly, 4 h following sham or pMCAO surgery, mice were anaesthetised using intraperitoneal administration of 150 mg/kg ketamine and 10 mg/kg xylazine. Animals were placed dorsal side onto a base plate, and thermostatically maintained at 37°C. A midline incision (approximately 30 mm) through the skin was made from the base of the abdomen to the top of the sternum. Vessels on the underside of the skin were cauterised, and an incision through the peritoneum was made at the bottom of the abdomen and extended to the base of the sternum. The ileum was isolated and placed onto a 3D-printed platform (serosa blood flow) or cut open to expose the lumen (villi blood flow), stabilised using a saline-soaked Kim wipe, and sealed using an imaging chamber plate. FITC-conjugated anti-mouse CD31 (390; eBioscience) or rhodamine-conjugated dextran and 5 μL of yellow-green microspheres (YG; Dia = 0.91 μ, SD = 0.017 μ, CV = 2%; Fluoresbrite; 9003-53-6) were administered intravenously to label the vasculature and detect blood flow, respectively. Imaging of the ileum via intravital microscopy was performed using an FVMPE-RS Multiphoton microscope (DM6000) at ×25 magnification (water; NA = 1.05) with Spectra-Physics InSight X3 laser and the Olympus FV315-SW software. A video recording of up to 33 s with a 0.03-s frame rate was taken across 3 fields of view (FOV) for each mouse. Microscopy videos were analysed using FIJI 1.53 by converting the videos into a hyperstack. To determine the rate of blood flow, the “Manual Tracking” plugin was used to calculate the velocity of the microbeads. In a blinded manner, a minimum of 10 microbeads was assessed for each FOV, and the results of the 3 FOV were averaged per mouse. Values were expressed as seconds per micrometre.

### Assessment of Cell Death via Flow Cytometry

At experimental endpoints, mice were culled via anaesthesia overdose and cervical dislocation. The intestinal tissue was dissected and separated approximately into duodenum, jejunum, and ileum (~10 cm each), as well as colon. Each segment was opened longitudinally along the mesenteric border, washed with sterile PBS, weighed, cut into 1-cm segments, and then incubated in EDTA solution (10% foetal calf serum (FCS, Life Technologies) in Ca^2+^ and Mg^2+^ free Hanks Balanced Salt Solution (HBSS) and 5 mM EDTA) for 45 min at 37°C at 120 rpm on a shaker. Next, the supernatant was filtered using a 70-µm filter and washed twice with PBS to remove EDTA. Single cells were treated with Fc Block (2.4G2; BD Pharmingen) and stained with APC-conjugated anti-EpCAM (G8.8; ThermoFisher), PE-conjugated anti-CD45 (30-F11; eBioscience), and BV421 Annexin V (BD Biosciences) in Annexin compatible buffer (2.5 mM CaCl_2_ in Ca^2+^ and Mg^2+^ free HBSS buffer) for 20 min on ice. To determine cell viability and count, 7-aminoactinomycin D (7-AAD; BioLegend) and 5 µL of counting beads (335925; BD) were added to the cells, respectively. Samples were processed using an LSR-Fortessa X20 (BD Biosciences), and the data were analysed using FlowJo 10.0.7 (Tree Star).

### Assessment of Cell Death Within the Epithelium of the Ileum

The ileum tissue was processed into Swiss-rolls, fixed overnight, embedded in paraffin wax, cut into 4-μm sections, and mounted onto slides as detailed above. To assess cell death by detection of fragmented DNA during apoptosis, tissue sections were stained with the terminal deoxynucleotidyl transferase dUTP nick end labelling (TUNEL) method in accordance with the Click-iT Plus TUNEL Assay protocol (Invitrogen, C10617). The sections were then counterstained for DAPI (Sigma-Aldrich) for 20 min at RT and coverslipped using DAKO fluorescence mounting medium (Agilent). A separate cohort of sections were incubated with rabbit anti-mouse cleaved caspase-3 (Casp3; Cell Signalling, 9661S) and Alexa Fluor 488-conjugated anti-mouse Ki67 (Invitrogen, 53-5698-82) overnight at 4°C. Sections were then stained with Alexa Flour 568-conjugated donkey anti-rabbit secondary antibody for 1 h at RT and counterstained with DAPI (Sigma-Aldrich). Imaging was performed with a Nikon A1R confocal microscope using ×40 and ×20 objective for TUNEL and Casp3, respectively. A total of 7 random FOV were captured for each mouse, and the resulting individual images were de-identified to facilitate blinded image analysis. The crypt and villus of the captured images were then sampled to yield 3 regions of interest (ROI) per intestinal sub compartment, and the total number of TUNEL-positive or Casp3-positive cells per total sampled crypt and villus were quantified.

### Whole-Genome RNA Sequencing of EpCAM^+^ Cells from the Small Intestine

RNA from epithelial cells was isolated using the RNeasy Mini kit as per manufacture protocol with minor modifications (Qiagen). One volume of 70% ethanol was added to the samples prior to transfer to RNeasy MinElute spin columns. Samples were centrifuged for 15 s at 8000*g*, and flow-through was discarded. A volume of 350 μL of Buffer RW1 was added to the samples and centrifuged for 15 s at 8000*g.* Flow-through was discarded before adding 80 μL of DNase I incubation mix (10 μL DNase I stock solution: 70 μL Buffer RDD). Samples were incubated for 15 min at RT. A volume of 350 μL of Buffer RW1 was added to the samples and centrifuged for 15 s at 8000*g*. The flow-through was discarded, and a volume of 500 μL Buffer RPE was added to the samples, followed by centrifugation for 15 s at 8000*g*. The flow-through was discarded. A volume of 500 μL of Buffer RPE was added to the samples and centrifuged for 15 s at 8000*g*. The flow-through was then discarded. The collection tube was replaced, and samples were centrifuged for 1 min at 8000*g*. The collection tube was replaced; 50 μL RNase-free water was added directly to the membrane of the column and centrifuged for 1 min at 8000*g* to elute the RNA. RNA samples isolated from epithelial cells from sham-operated and post-stroke mice were stored at −80°C.

The quality of the extracted RNA was tested using an RNA 6000 Pico kit on the Agilent 2100 Bioanalyser, and only samples containing distinguishable 18S and 28S peaks on the electropherogram were included in the study. An RNA cleavage approach was carried out to remove ribosomal RNA. Library construction and cDNA synthesis were performed using the Trio RNA-sequencing library preparation kit (protocol M01440v2, 2017; Tecan). Manual denaturing and on-board clustering were carried out using 70 pM of library pool containing 1% PhiX (protocol 1000000109376 v3, 2020; Illumina). Sequencing was carried out on a NextSeq 2000 (Illumina) with P3 reagents and paired-end reading of 100 base pairs. Approximately 35 million reads were sequenced for each sample.

To process and analyse the RNA sequencing data, the interactive web tool Degust 4.1.1 was used by the Monash Bioinformatics Platform (MBP) in a blinded manner [27]. MultiQC 1.10.1 located within Degust was used to visualise and track the statistical data obtained during the preparation of the gene library (https://multiqc.info/). Firstly, the quality of the raw reads in the gene library was assessed with FastQC. Gene libraries were then aligned to the *Mus* *musculus* reference genome (GRCm38) using STAR through the rnasik 1.5.4 pipeline, whereby approximately 82% of the aligned data were considered as uniquely mapped reads [28]. Genomic features assigned (approximately 40%) to the library were quantified using featureCounts [29]. The following general alignment (BAM) files were indexed using SAMtools [30]. The quality of the aligned reads was then checked using Picard.

In Degust, the Limma-Voom method was used to calculate differentially expressed (DE) genes between the sham and pMCAO groups. Samples were corrected for batch effects across different days of tissue collection. As quality control measures, the *p*-value distribution, library size, and gene expression (counts per million (CPM)) were compared between samples. Differentially expressed genes were considered significant if the false discovery rate (FDR) was ≤ 0.05 and absolute logFC = 1. DE genes were further analysed to study gene ontologies (GO), gene enrichment, and protein-protein interactions. Over-represented GO terms associated with specific biological processes (BP) were obtained using The Gene Ontology Consortium database (PANTHER 16.0 Overrepresentation test; Fisher’s exact test; FDR < 0.05 by the Benjamin and Hochberg method) [31–33]. Biological pathway analysis was carried out using the Kyoto Encyclopedia of Genes and Genomes (KEGG) database [34, 35].

### Intestinal Organoids

The distal third (9 cm from caecum) of the small intestine was isolated from wild-type mice. Epithelial crypts were isolated according to established procedures [36], but scraping of the coverslip was avoided to increase the recovery of crypt material. Crypts were resuspended in Matrigel and grown in a small intestinal culture medium. Established organoids were passaged using mechanical disruption and replated for use at passage 2 or 3. For assays, organoids were passaged using a P1000 tip and suspended in Matrigel to achieve 120–150 fragments per well of a 48-well plate. In total, 20 μL of Matrigel with organoid fragments was seeded per well and fed with media every 2 days until organoids formed budding structures at day 4. Organoids were treated with noradrenaline (0.1, 1, 10, or 100 μM) or vehicle (PBS), on day 4 for 5 h, before being harvested. Brightfield images were collected at 0 h, 2.5 h, and 5 h (EVOS FL Imaging System). At 5 h, supernatant and organoids were collected from quadruplicate wells per concentration, and additional triplicate wells were stained with EarlyTox assay and imaged using an ImageXpress PICO system, followed by fixation (5% formalin). MetaXpress software was used to measure the total number of dead cells per well for each condition. Fixed organoids were stained with DAPI and images were captured using the EVOS M7000 Imaging system. Triplicate measurements from separate wells within experiments were averaged, and the averages of these compared between experiments. Organoid experiments were completed with four independent biological replicates.

### Statistical Analysis

The data were analysed using the GraphPad Prism software. Normality testing was done using the Shapiro-Wilk test. Parametric data were compared by unpaired two-tailed Student’s *t*-test or two-way ANOVA with the Holm-Šídák multiple comparisons test. Non-parametric data were compared by the unpaired two-tailed Mann-Whitney *U*-test or one-way ANOVA with the Kruskal-Wallis *H* test. Organoid experiment was analysed using the Friedman test. Outliers were assessed using the GraphPad outlier calculator (Grubbs’ test; Alpha = 0.05). Graphical data are shown as mean ± standard error of the mean (SEM). Statistical significance is accepted at *p* < 0.05.

## Results

### Stroke-Induced Gut Permeability Is Independent of Intestinal Perfusion and Inflammation

**Fig. 1:**
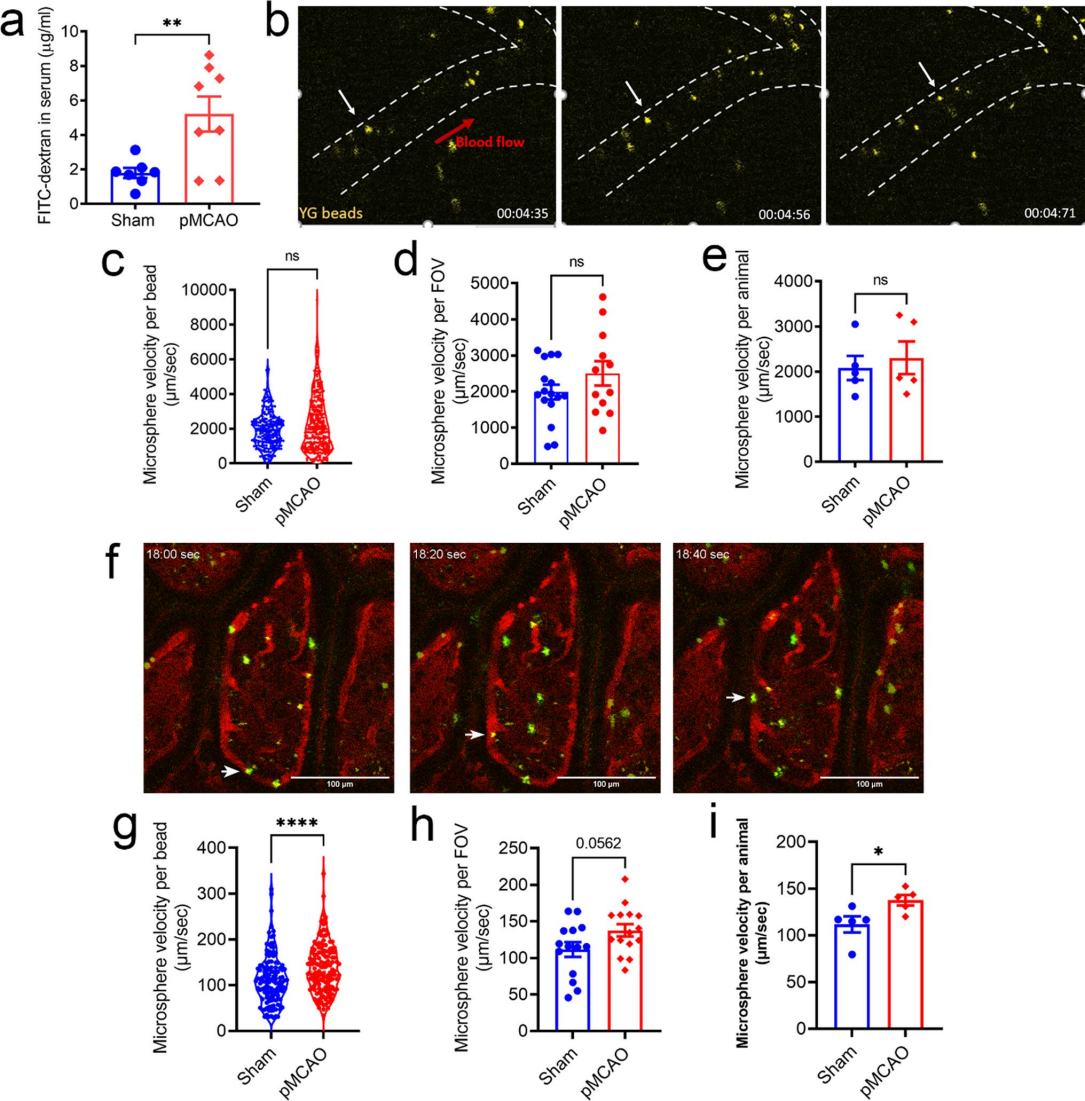
Stroke-induced gut permeability is not contributed by reduced intestinal perfusion. **a** Quantitative analysis of FITC-dextran concentration in the serum (µg/mL; sham: *n* = 7; pMCAO: *n* = 8). ** *p* <0.01 versus sham by unpaired two-tailed Student’s *t*-test. The ileal serosal (**b–e**) and villus microvasculature (**f–i**) blood flow in mice that underwent sham or permanent middle-cerebral occlusion (pMCAO) surgery was measured via *in vivo* imaging. **b** Representative images of microbeads (yellow; white arrow) in the blood vessel of ileal serosa (denoted by the white dotted lines). Quantification of individual microbead velocity was assessed, and each data point represents **c** the velocity per bead assessed, **d** the average value per field of view (FOV), or **e** the average value per animal. **f** Representative images of microbeads (green; white arrow) in the blood vessel of ileal villi. Quantification of individual microbead velocity was assessed, and each data point represents **g** the velocity per bead assessed, **h** the average value per field of view (FOV), or **i** the average value per animal (*n* = 5 animals per group). ns denotes not statistically significant, *p <0.05, ****p <0.0001 versus sham by assessed by unpaired two-tailed Mann-Whitney *U*-test in (**c,** **e, g, i**) and unpaired two-tailed Student’s *t*-test in (**d, h**). Data are shown as mean ± SEM

Clinically, fewer than 15% of ischaemic stroke patients are given thrombolytic agents to restore blood supply to the brain; though some may experience spontaneous reperfusion, many do not necessarily undergo reperfusion [37]. For this reason, we utilised the permanent middle cerebral artery occlusion (pMCAO) model of experimental stroke to examine whether post-stroke mice exhibit gut barrier impairment, and further explored the possible mechanisms of this post-stroke gut pathology. Using the well-characterised *in vivo* assay [6], we found a significantly higher concentration of the orally gavaged FITC-dextran in the serum of pMCAO mice compared to sham-operated mice at 5 h following surgery (Fig. 1a). This indicates that gut permeability is elevated during the acute stages of this model of experimental stroke. This finding is consistent with our previous study where a milder model of experimental stroke was utilised to reveal that the ileal tissue was most affected after cerebral ischaemia-reperfusion injury [6]. Informed by this, we focused this study on the effect of non-reperfused cerebral ischaemia on the ileum and investigated the potential mechanisms for post-stroke gut permeability. Altered intestinal perfusion and mucosal inflammation have previously been shown to contribute to gut leakiness [38, 39]. At 5 h following surgery, we found comparable blood flow velocity at the ileum serosa (Fig. 1b–e), whilst villi microvasculature demonstrated enhanced blood velocity post-stroke (Fig. 1f–i). These findings suggest hypoperfusion is unlikely to be contributing factor to acute gut permeability after stroke. In addition, we detected similar expression levels of inflammatory markers (Supplemental Fig. 1), and found no histological differences between the ileum of sham-operated (Supplemental Fig. 2a) and post-stroke mice (Supplemental Fig. 2b). Moreover, the length of intestine (Supplemental Figure 2c) and ileal villi length (Supplemental Fig. 2d) were similar between pMCAO and sham-operated mice at this timepoint. Taken together, our data indicates that experimental stroke does not lead to altered intestinal perfusion, inflammation, or macroscopic structural changes at the timepoint of elevated gut permeability.

**Fig. 2:**
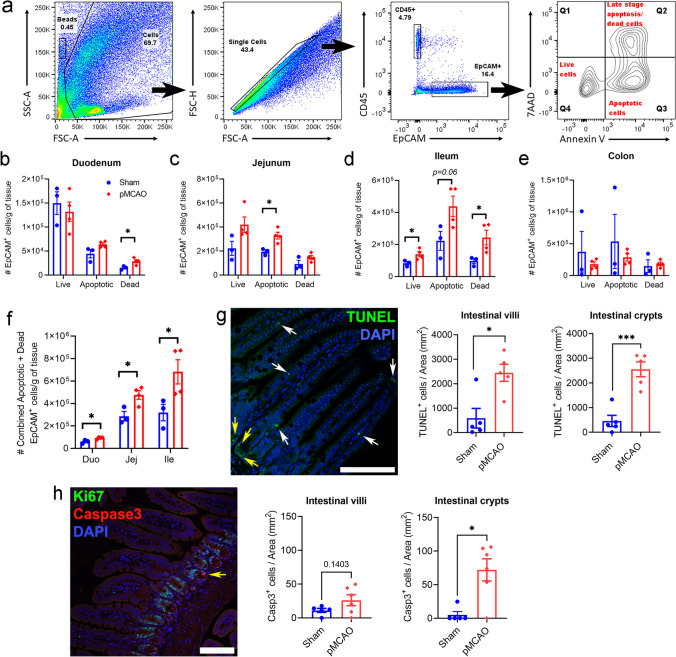
Elevated intestinal epithelial cell death and permeability at 5 h after stroke. **a** Gating strategy for assessment of intestinal epithelial cell death via flow cytometry. Quantitative analysis of live, apoptotic, and dead epithelial cells in the duodenum (**b**), jejunum (**c**), ileum (**d**), and colon (**e**). **f** Quantitative analysis of combined apoptotic and dead epithelial cells in small intestinal regions (sham: *n* = 3; pMCAO: *n* = 4; *p < 0.05 versus sham by unpaired two-tailed Student’s *t*-test). **g** Representative image of TUNEL staining in the ileum (scale bar = 100 µm; white arrows denote TUNEL^+^ cells in the villi; yellow arrows denote TUNEL^+^ cell in crypt) and quantitative analysis of TUNEL^+^ cells per area (mm^2^) in the intestinal villi and crypts (villi: *n* = 5 for both groups). *p < 0.05 versus sham by unpaired two-tailed Mann-Whitney *U* test; ***p < 0.001 versus sham by unpaired two-tailed Student’s *t*-test. **h** Representative image of cleaved caspase-3 (Casp3) staining in the ileum (scale bar = 100 µm; yellow arrow denotes Casp3^+^ cell in crypt). Quantitative analysis of Casp3^+^ cells per area (mm^2^) in the intestinal villi (sham: *n* = 5; pMCAO: *n* = 6; unpaired two-tailed Student’s *t*-test) and crypts (sham: *n* = 5; pMCAO: *n* = 6; *p < 0.05 versus sham by unpaired two-tailed Mann-Whitney *U* test. Data are shown as mean ± SEM

### Elevated Intestinal Epithelial Cell Turnover After Stroke

We next tested the hypothesis that post-stroke gut permeability is attributed to excessive intestinal epithelial cell death triggered by cerebral ischaemia. We performed flow cytometry on various gut regions of sham-operated and post-stroke mice to examine the expression of markers known to detect apoptotic (Annexin V) and dead cells (7AAD). Using published gating strategies (Fig. 2a), we defined live cells as EpCAM^+^7AAD^-^Annexin V^-^ (Q4); apoptotic cells as EpCAM^+^7AAD^-^Annexin V^+^ (Q3); and late-stage apoptotic/dead cells as EpCAM^+^7AAD^+^Annexin V^-^ (Q2). We detected significantly elevated apoptotic or dead epithelial cells in the duodenum (Fig. 2b), jejunum (Fig. 2c), and ileum (Fig. 2d) in post-stroke mice compared to their sham-operated counterparts. This stroke-induced intestinal epithelial cell death was not found in the colon (Fig. 2e). Given that the post-stroke ileum demonstrated the greatest number of combined apoptotic and dead epithelial cells (Fig. 2f), we next performed TUNEL and immunofluorescence of cleaved caspase-3 (Casp3) to investigate whether these cells were located on ileal proliferative cells (confined to crypts) or differentiated cells (predominantly occupying the villi). At 5 h after surgery, we found significant increase of TUNEL^+^ cells in the epithelial villi and crypts in the ileum of post-stroke mice compared to their sham-operated counterparts (Fig. 2g). Moreover, we observed cells undergoing apoptosis in the intestinal crypt after stroke, a phenomenon rarely seen in their sham-operated counterparts (Fig. 2h).

**Fig. 3:**
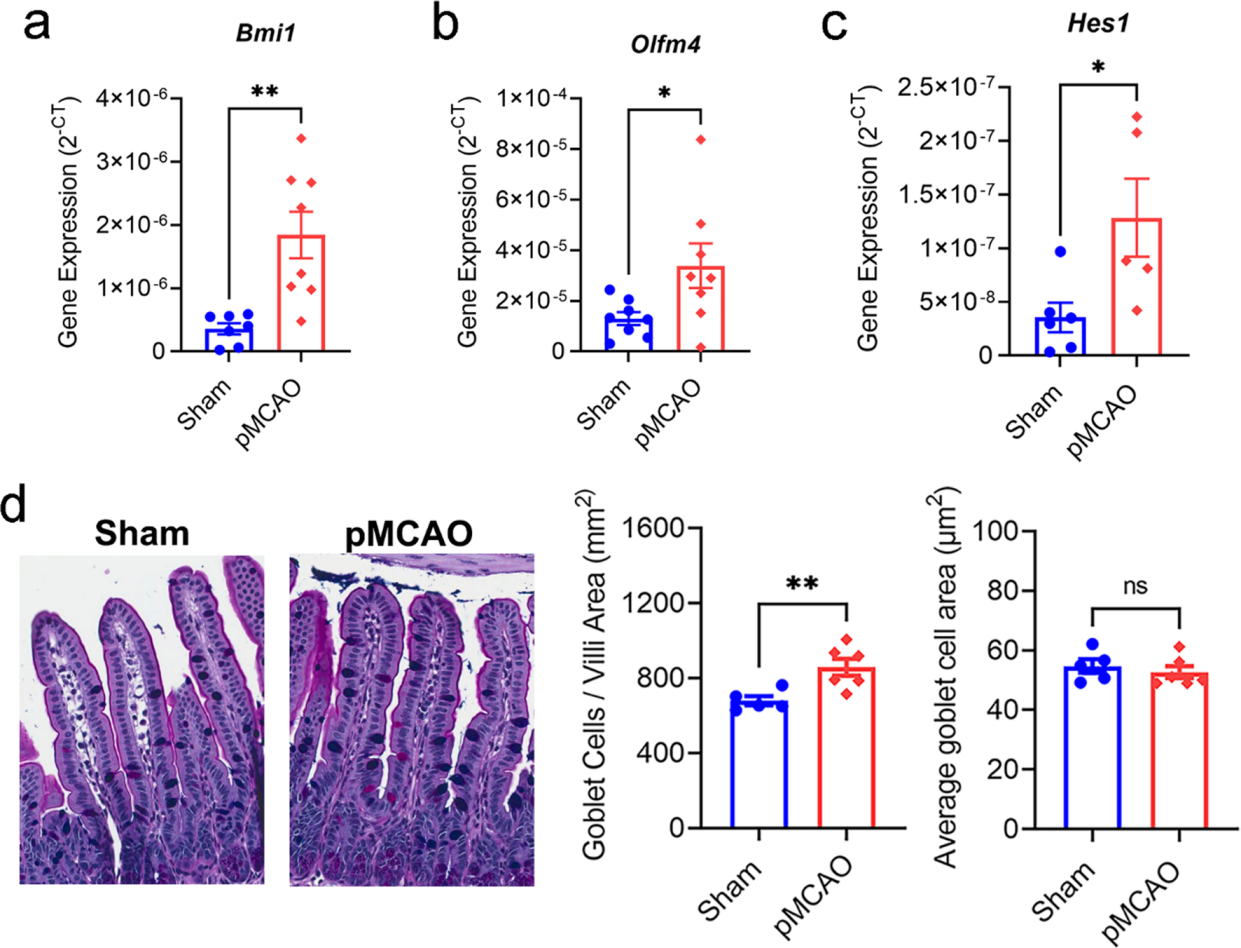
Altered stem and progenitor cell gene signatures at 5 h after stroke. Gene expression analysis of *Bmi1* (**a**), *Olfm4* (**b**), and *Hes1* (**c**) in epithelial cells of the whole small intestine via qPCR. (**d**) Representative images of goblet cells in the ileum tissue via PAS-Alcian blue staining, quantitative analysis of number of goblet cells per area (mm^2^), and average goblet cell area (µm^2^; sham: *n* = 5; pMCAO: *n* = 6). *p < 0.05, **p < 0.01 versus sham by unpaired two-tailed Student’s* t*-test. Each data point represents the average value of one animal. Data are shown as mean ± SEM

In additional to the novel findings of increased intestinal epithelial cell death in the ileum of pMCAO mice at 5 h, we found elevated expression of markers of stem and progenitor cells, including *Bmi1* (Fig. 3a), *Olfm4* (Fig. 3b), and *Hes1* (Fig. 3c) [40–43]. Furthermore, we detected a greater number of mucin-producing goblet cells, which were morphologically similar to the sham cohort, in the small intestine at 5 h post-stroke (Fig. 3d). Conceivably, the upregulation of markers for stem and progenitor cells, and elevated goblet cell numbers are to counteract the enhanced intestinal cell death at 5 h after stroke. Therefore, we proposed that these changes are part of an adaptive host response to robustly replace dying epithelial cells to prevent further gut leakiness after stroke.

**Fig. 4:**
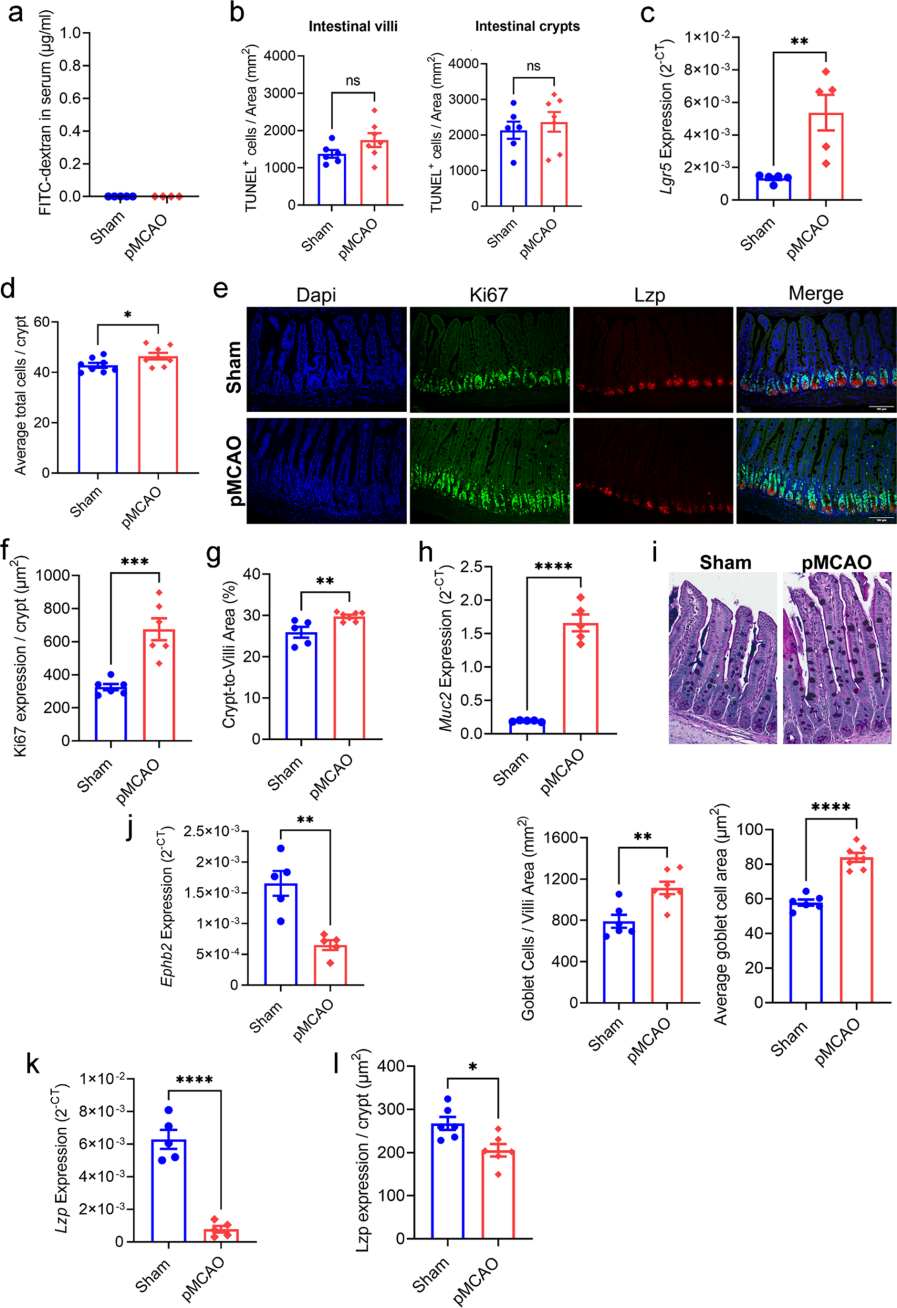
Crypt and goblet cell hyperplasia at 24 h after stroke.** a** Quantitative analysis of FITC-dextran concentration in the serum (µg/mL; sham: *n* = 5; pMCAO: *n* = 4). **b** Quantitative analysis of TUNEL^+^ cells per area (mm^2^) in the intestinal villi and crypts (sham: *n* = 6; pMCAO: *n* = 7 for both groups). Unpaired two-tailed Student’s *t*-test. **c** Gene expression analysis of *Lgr5* in epithelial cells of the whole small intestine via qPCR (*n* = 6 per group). **p < 0.01 versus sham by unpaired two-tailed Mann-Whitney *U* test. **d** Average number of cells per ileal crypt (*n* = 8 per group). *p < 0.05 versus sham by unpaired two-tailed Student’s *t*-test. **e** Representative image of Ki67^+^ and Lzp^+^ cells in the intestinal of sham-operated and post-stroke mice at 24 h (scale bar = 100 µm). **f** Quantitative analysis of Ki67 expression per crypt at 24 h (*n* = 6 per group). ***p < 0.001 versus sham by unpaired two-tailed Student’s *t*-test. **g** Ileal crypt to villi area ratio (sham: *n* = 5; pMCAO: *n* = 7). **p < 0.01 versus sham by unpaired two-tailed Student’s* t*-test. **h** Gene expression analysis of *Muc2* in epithelial cells of the whole small intestine via qPCR (*n* = 5 per group). ****p < 0.0001 versus sham by unpaired two-tailed Student’s *t*-test. **i** Representative images of goblet cells in the ileum tissue via PAS-Alcian blue staining, quantitative analysis of number of goblet cells per area (mm^2^), and average goblet cell area (µm^2^; sham: *n* = 6; pMCAO: *n* = 7). **p < 0.01, ****p < 0.0001 versus sham by unpaired two-tailed Student’s *t*-test. Gene expression analysis of *Ephb2* (**j**) and *Lzp* (**k**) in epithelial cells of the whole small intestine via qPCR (*n* = 5 for both groups). **p < 0.01, ****p < 0.0001 versus sham by unpaired two-tailed Student’s *t*-test. **l** Quantitative analysis of Lzp expression per crypt at 24 h (*n* = 6 per group). *p < 0.05 versus sham by unpaired two-tailed Student’s *t*-test. Each data point represents the average value of one animal. Data are shown as mean ± SEM

In support of this notion, we found post-stroke gut permeability was no longer observed at 24 h following stroke onset (Fig. 4a), a finding consistent with our previous report using a milder model of experimental stroke [6]. Additionally, stroke-induced cell death in the ileum was no longer observed at this timepoint (Fig. 4b). Instead, at 24 h after stroke, we observed significantly higher epithelial expression of stem cell marker *Lgr5* (Fig. 4c) [44], and a greater number of cells within intestinal crypts (Fig. 4d) which were highly expressing the proliferative marker Ki67 (Fig. 4e, f). Furthermore, the elevated crypt–villi area ratio at 24 h after stroke is indicative of enhanced epithelial cell proliferative capacity in the intestinal crypts (Fig. 4g). Next, we investigate if the goblet cell population, important for maintaining the mucus barrier, was altered 24 h after stroke. We observed increased *Muc2* expression (Fig. 4h), and correspondingly greater number and size of goblet cells (Fig. 4i) in the post-stroke animals. In contrast, there was reduced expression of *Ephb2* (Fig. 4j) and *Lzp* (Fig. 4k) as well as Lzp staining (Fig. 4e, l) in post-stroke mice compared to sham-operated controls, indicating there were less transit amplifying progenitor cells and antibacterial Paneth cells after stroke, respectively. Collectively, our findings suggest that stroke promotes intestinal epithelial cell death and activates stem and progenitor cells to stimulate proliferation and crypt hyperplasia, possibly as a host compensatory mechanism to adapt to impaired gut barrier integrity following brain injury.

### Transcriptomic Changes in Enterocytes After Stroke

**Fig. 5:**
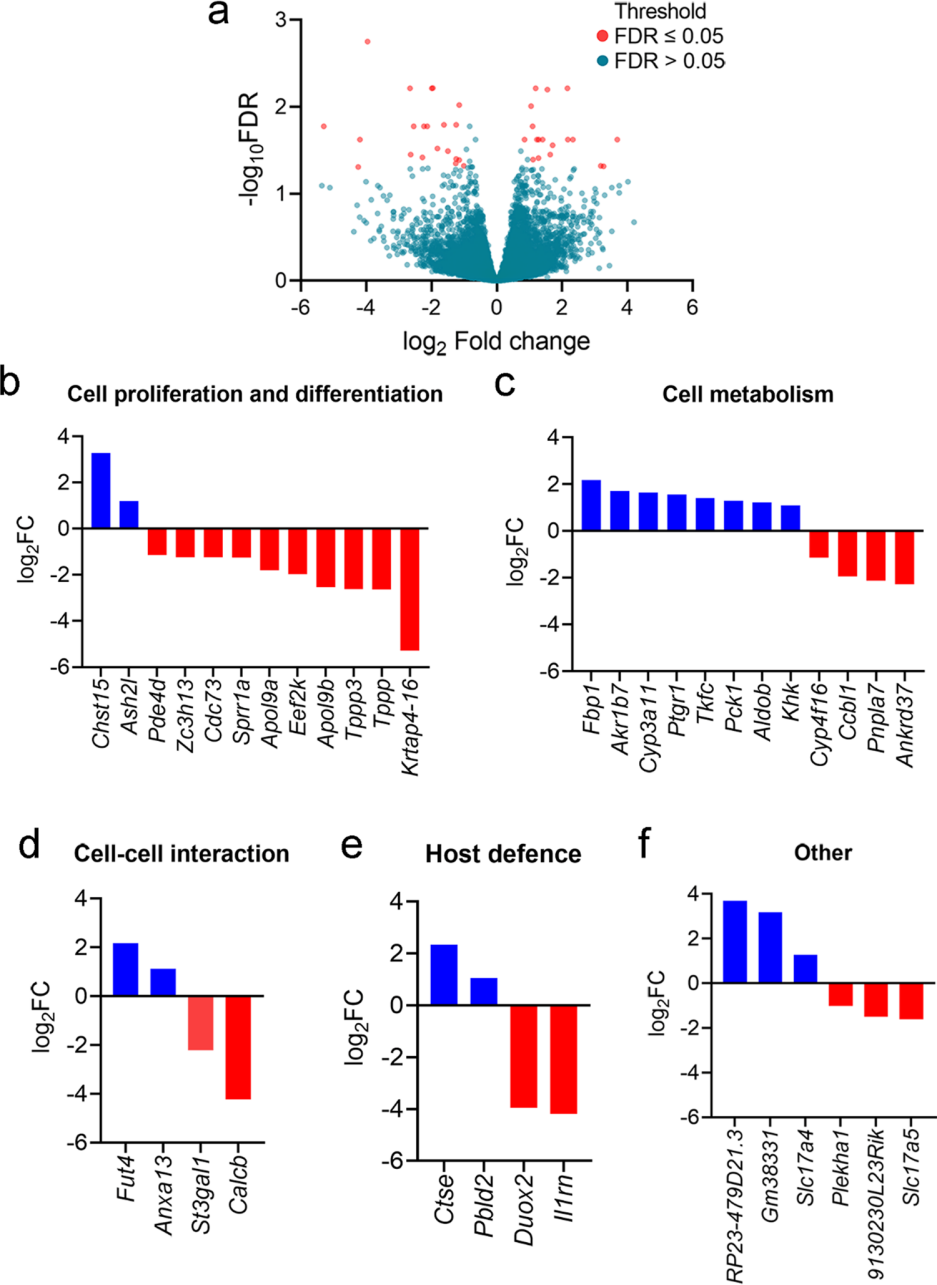
Enhanced fructose metabolism in post-stroke enterocytes. **a** Volcano plot showing differentially expressed genes in in epithelial cells from the whole small intestine at 24 h (sham: *n* = 7; pMCAO: *n* = 6). The plot shows false discovery rate (-log_10_FDR) versus log_2_ fold change (log_2_ FC). Differentially expressed genes are shown in red (FDR of ≤ 0.05, absolute log FC = 1, Voom/limma). Log2 fold change of genes related to **b** cell proliferation and differentiation, **c** cell metabolism, **d** cell-cell interaction, **e** host defence, and **f** other (sham: *n* = 7; pMCAO: *n* = 6)

To the best of our knowledge, stroke-induced intestinal cell death followed by epithelial proliferation has not been reported previously. To gain insight into the impact of stroke on the molecular machinery of the intestinal epithelium proliferation, we performed RNA sequencing on the EpCAM^+^ epithelial cell population from the small intestine of sham-operated and post-stroke mice at 24 h. We discovered 38 significant differentially expressed (DE) genes in the epithelial cells of pMCAO animals compared to the sham-operated group (Fig. 5a). We have listed these genes in the order of significance in Table 1, and functionally categorised them into “Cell proliferation and differentiation” (Fig. 5b), “Metabolism” (Fig. 5c), “Cell-cell interaction” (Fig. 5d), “Host defence” (Fig. 5e), and “Others” (Fig. 5f). Next, the biological relevance of the 38 DE genes in epithelial cells altered by stroke was assessed using gene ontology (GO) analysis. Over-represented GO terms associated with biological processes (BP) were determined using PANTHER [31–33]. Genes with no identifiable protein counterpart were excluded from the analysis, and these were *RP23-479D21* and *Gm38331*. It was found that all 14 of the GO terms were associated with carbohydrate metabolism, with the most significantly associated BP being the “fructose metabolic process” (Table 2). Next, the biological pathway that these DE genes participate in was studied using the Kyoto Encyclopedia of Genes and Genomes (KEGG) database [34, 35]. Six biological pathways were identified, and they were all associated with cell metabolism (Table 3). Specifically, KEGG revealed that the pathway most significantly associated with the DE genes was the “fructose and mannose metabolism” pathway. Given dietary fructose improves the survival of intestinal cells and nutrient absorption [45], our findings reveal that although stroke may dysregulate *de novo* epithelial cell replenishment, the adaptive hyperactivation of fructose metabolism within pre-existing epithelial cells may act to enhance cell survivability and ameliorate intestinal barrier disruption.

**Table 1:** Differentially expressed genes in EpCAM^+^ epithelial cells

Gene name	Protein names	Stroke fold change relative to sham (logFoldChange)	Average expression	False discovery rate
*Duox2*	Dual oxidase 2	−3.96	5.66	1.77E−03
*Ccbl1*	Glutamine transaminase K	−1.96	5.32	6.08E−03
*Tppp*	Tubulin polymerization promoting protein	−2.66	4.88	6.11E−03
*Eef2k*	Eukaryotic elongation factor−2 kinase	−1.99	5.25	6.11E−03
*Ash2l*	ASH2 like histone lysine methyltransferase complex subunit	1.19	4.82	6.11E−03
*Fut4*	Fucosyltransferase 4	2.16	2.87	6.11E−03
*Ptgr1*	Prostaglandin reductase 1	1.55	5.02	6.33E−03
*Pde4d*	Phosphodiesterase 4D, cAMP specific	−1.15	6.23	9.53E−03
*Pbld2*	Phenazine biosynthesis-like protein domain containing 2	1.05	5.66	9.80E−03
*Zc3h13*	Zinc finger CCCH type containing 13	−1.24	7.53	1.61E−02
*Slc17a5*	Solute carrier family 17 (anion/sugar transporter), member 5	−1.62	6.07	1.61E−02
*Krtap4-16*	Keratin associated protein 4-16	−5.29	−1.12	1.67E−02
*St3gal1*	ST3 beta-galactoside alpha-2,3-sialyltransferase 1	−2.23	5.92	1.67E−02
*Apol9b*	Apolipoprotein L 9b	−2.54	5.40	1.67E−02
*Pnpla7*	Patatin-like phospholipase domain containing 7	−2.13	3.82	1.67E−02
*Khk*	Ketohexokinase	1.10	5.83	1.67E−02
*Ctse*	Cathepsin E	2.33	1.74	2.37E−02
*Fbp1*	Fructose bisphosphatase 1	2.17	2.07	2.37E−02
*Tkfc*	Triokinase, FMN cyclase	1.40	6.21	2.37E−02
*Il1rn*	Interleukin 1 receptor antagonist	−4.19	−1.68	2.37E−02
*Pck1*	Phosphoenolpyruvate carboxykinase 1, cytosolic	1.29	7.87	2.37E−02
*Aldob*	Aldolase B, fructose-bisphosphate	1.23	10.53	2.37E−02
*RP23-479D21.3*	N/A	3.69	−1.10	2.37E−02
*Akr1b7*	Aldo-keto reductase family 1, member B7	1.71	4.31	2.77E−02
*Apol9a*	Apolipoprotein L 9a	−1.82	6.32	3.01E−02
*9130230L23Rik*	N/A	−1.50	5.55	3.22E−02
*Cyp3a11*	Cytochrome P450, family 3, subfamily a, polypeptide 11	1.63	7.26	3.53E−02
*Tppp3*	Tubulin polymerization-promoting protein family member 3	−2.64	3.11	3.53E−02
*Ankrd37*	Ankyrin repeat domain 37	−2.28	3.76	3.81E−02
*Slc17a4*	Solute carrier family 17 (sodium phosphate), member 4	1.28	6.93	3.89E−02
*Cdc73*	Cell division cycle 73, Paf1/RNA polymerase II complex component	−1.25	6.15	3.97E−02
*Anxa13*	Annexin A13	1.11	6.45	4.03E−02
*Cyp4f16*	Cytochrome P450, family 4, subfamily f, polypeptide 16	−1.15	6.37	4.07E−02
*Sprr1a*	Small proline-rich protein 1A	−1.26	3.90	4.48E−02
*Gm38331*	Predicted gene, 38331	3.18	−1.85	4.77E−02
*Plekha1*	Pleckstrin homology domain containing, family A (phosphoinositide binding specific) member 1	−1.01	7.80	4.77E−02
*Chst15*	Carbohydrate sulfotransferase 15	3.27	0.94	4.84E−02
*Calcb*	Calcitonin-related polypeptide, beta	−4.24	−1.38	4.91E−02

**Table 2:** Gene ontology terms associated with differentially expressed genes in EpCAM^+^ intestinal epithelial cells

Gene ontology (biological process)	Gene ontology ID	No. of associated genes	Fold enrichment	-log_10_ False DiscoveryRate
Fructose metabolic process	GO:0006000	4	> 100	3.97881
Fructose catabolic process to hydroxyacetone phosphate and glyceraldehyde-3-phosphate	GO:0061624	3	> 100	3.56225
Glycolytic process through fructose-1-phosphate	GO:0061625	3	> 100	3.46471
Monosaccharide metabolic process	GO:0005996	6	24.76	3.40121
Pyruvate metabolic process	GO:0006090	5	42.45	3.37572
Fructose catabolic process	GO:0006001	3	> 100	3.34008
Hexose metabolic process	GO:0019318	6	28.3	3.18709
Glyceraldehyde-3-phosphate metabolic process	GO:0019682	3	> 100	3.14388
Carbohydrate metabolic process	GO:0005975	7	10.1	2.02457
Hexose catabolic process	GO:0019320	3	89.14	1.92445
Monosaccharide catabolic process	GO:0046365	3	77.51	1.80134
Hexose biosynthetic process	GO:0019319	3	63.67	1.60206
Metabolic process	GO:0008152	25	1.99	1.40782
Monosaccharide biosynthetic process	GO:0046364	3	52.44	1.4034

**Table 3:** Biological pathway analysis of differentially expressed genes in EpCAM^+^ intestinal epithelial cells via KEGG

Pathway name	Pathway ID	Adjusted *p*-value	-log10 of adjusted *p*-value	Proteins
Fructose and mannose metabolism	KEGG:00051	6.40E−07	6.193709446	KHK, FBP1, TKFC, ALDOB, AKR1B7
Metabolic pathways	KEGG:01100	0.004711851	2.326808419	KYAT1, FUT4, PDE4D, ST3GAL1, KHK, FBP1, TKFC, PCK1, ALDOB, AKR1B7, CYP3A11
Glycolysis/gluconeogenesis	KEGG:00010	0.007012163	2.154148009	FBP1, PCK1, ALDOB
Pentose phosphate pathway	KEGG:00030	0.022860201	1.640919951	FBP1, ALDOB
Carbon metabolism	KEGG:01200	0.022860201	1.640919951	FBP1, TKFC, ALDOB
AMPK signalling pathway	KEGG:04152	0.022860201	1.640919951	EEF2K, FBP1, PCK1

#### Stroke-Induced Intestinal Epithelial Cell Turnover Is Independent of Microbiota

**Fig. 6:**
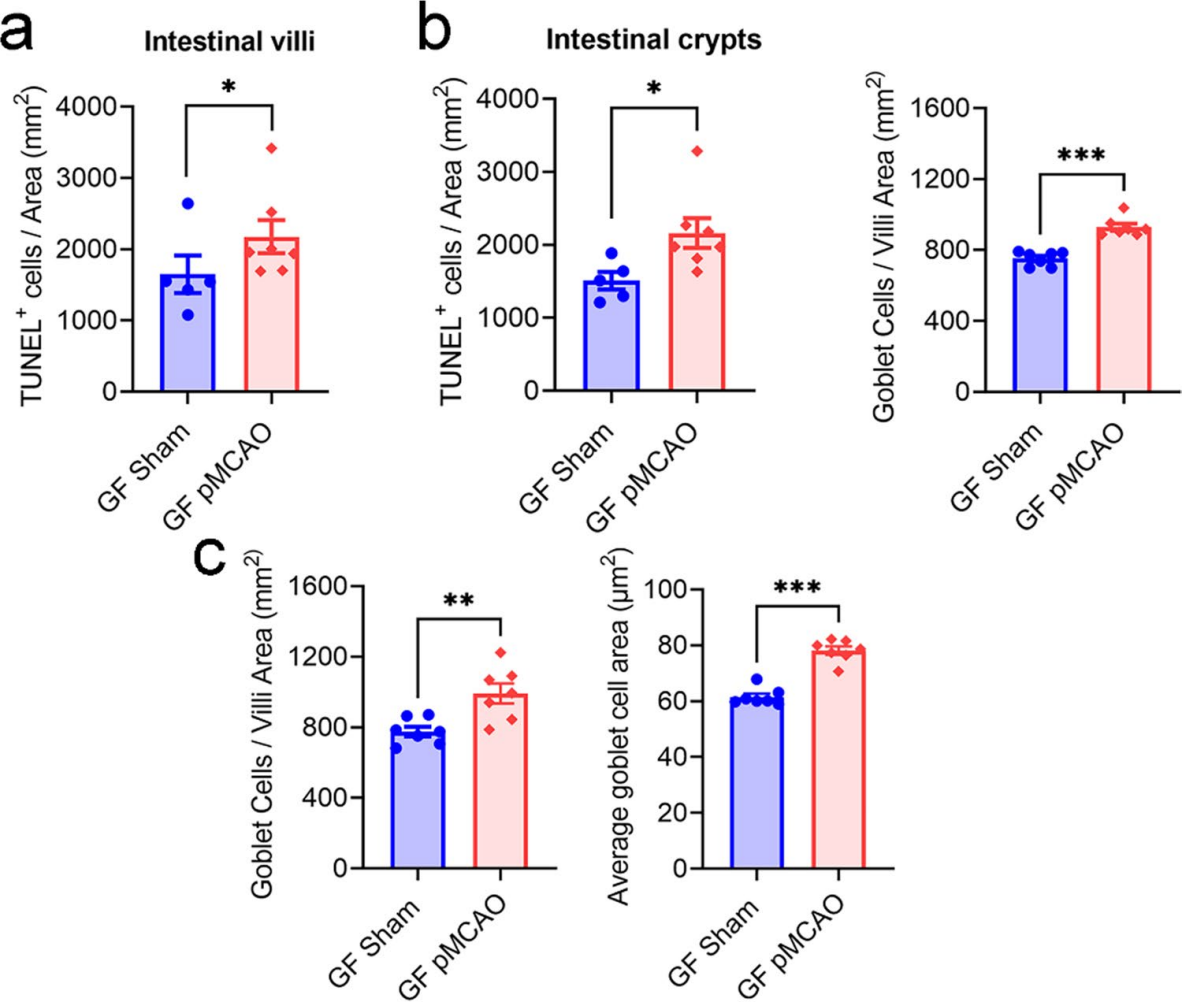
Stroke-induced intestinal epithelial cell death is independent of microbiota. Germ-free (GF) mice of C57BL/6J background underwent sham or permanent middle-cerebral occlusion (pMCAO) surgery. **a** Quantitative analysis of TUNEL^+^ cells per area (mm^2^) in the intestinal villi and crypts at 5 h (sham: *n* = 5; pMCAO: *n* = 7). *p < 0.05 versus sham by unpaired two-tailed Mann-Whitney *U* test. **b** Quantitative analysis of number of goblet cells per area (mm^2^) at 5 h (*n* = 7 for both groups). ***p < 0.001 versus sham by unpaired two-tailed Mann-Whitney *U* test. **c** Quantitative analysis of number of goblet cells per area (mm^2^) at 24 h (*n* = 7 for both groups). **p < 0.01 versus sham by unpaired two-tailed Student’s *t*-test. Mean goblet cell area (µm^2^; sham: *n* = 6; pMCAO: *n* = 7). ***p < 0.001 versus sham by unpaired two-tailed Mann-Whitney *U* test. Each data point represents the average value of one animal. Data are shown as mean ± SEM

Microbial dysbiosis is evident after stroke, and it has been suggested to promote gut barrier disruptions and worsening of stroke outcomes [23, 46, 47]. To test if intestinal epithelial cell death after stroke is influenced by microbial changes, we examined the ileum of sham-operated and post-stroke mice raised in a germ-free (GF) facility. Notably, bacterial colonisation is fundamental and indispensable for normal immunity and intestinal homeostasis; therefore, it is not our intention to compare SPF vs GF animals. Instead, we have utilised GF animals in this study to investigate if cerebral ischaemia elicits direct changes to the gut epithelium dependent of post-stroke dysbiosis. Our data revealed that the stroke-induced augmentation of intestinal epithelial cell death at 5 h in the SPF mice (see Fig. 1) was also observed in the GF animals (Fig. 6a). Furthermore, the post-stroke elevation of goblet cell numbers at 5 h (Fig. 6b) and 24 h (Fig. 6c) was evident in GF mice. These findings indicated that stroke-induced intestinal epithelial cell death and goblet cell hyperplasia are not influenced by post-stroke microbial dysbiosis.

### Post-Stroke Gut Permeability and Cell Death Is Mediated by Sympathetic Signalling

**Fig. 7:**
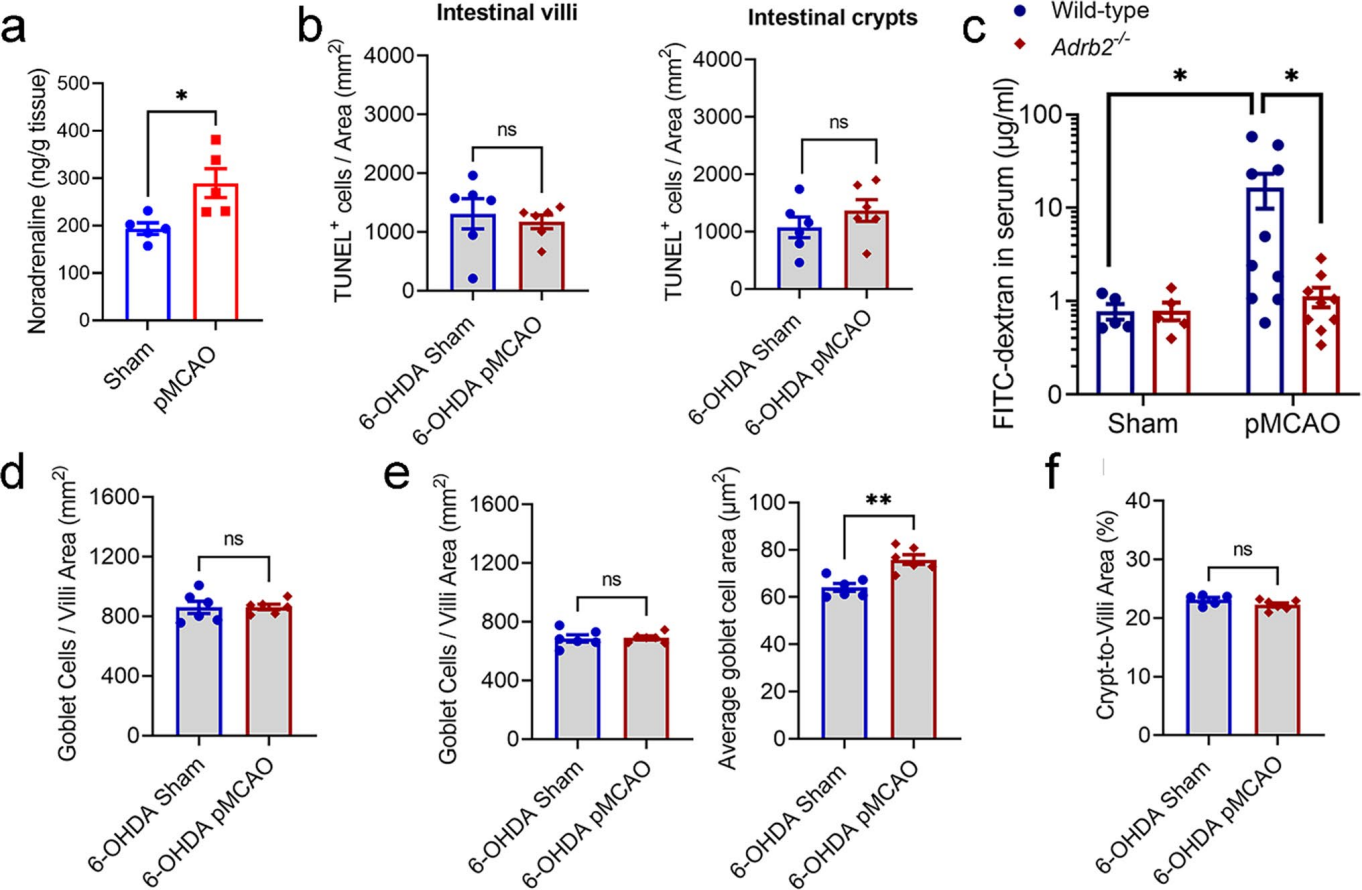
Gut permeability and cell death is mediated by sympathetic signalling. **a** The ileum tissue was isolated for the quantification of noradrenaline level. Mice were treated with 6-hydroxydopamine (6-OHDA; 100 mg/kg i.p) 3 days prior to surgery. **b** Quantitative analysis of TUNEL^+^ cells per area (mm^2^) in the intestinal villi and crypts at 5 h (*n* = 6 for both groups). Unpaired two-tailed Student’s *t*-test. **c** Wild-type and *Adrb2-/-* mice underwent sham or permanent middle-cerebral occlusion (pMCAO) surgery. Quantitative analysis of FITC-dextran concentration in the serum at 5 h (µg/mL; sham: *n* = 7; pMCAO: *n* = 8). *p <0.05 one-way ANOVA with the Kruskal-Wallis *H* test. **d** Quantitative analysis of number of goblet cells per area (mm^2^) at 5 h (*n* = 6 for both groups). Unpaired two-tailed Student’s *t-*test. **e** Quantitative analysis of number of goblet cells per area (mm^2^) and mean goblet cell area (µm^2^) at 24 h (*n* = 6 for both groups). **p < 0.01 versus sham by unpaired two-tailed Student’s *t*-test. **f** Ileal crypt to villi area ratio at 24 h (sham: *n* = 5; pMCAO: *n* = 6). Unpaired two-tailed Student’s *t*-test. Each data point represents the average value of one animal. Data are shown as mean ± SEM

The autonomic nervous system is intimately involved in the regulation of intestinal epithelium turnover processes, as denervation of either sympathetic or parasympathetic nerves to the intestine alters intestinal epithelial cell proliferation [19]. Therefore, we next investigated if activation of the SNS, as often observed in experimental [48] and clinical [49–53] stroke, is responsible for the increased intestinal cell death, elevated gut barrier leakiness, and crypt and goblet cell hyperplasia. Indeed, we observed an elevated amount of noradrenaline (NA) in the ileal tissue at 5 h after stroke (Fig. 7a). Stroke-induced epithelial cell death and apoptosis in the ileum were completely inhibited by the specific pharmacological denervation of peripheral neuronal terminals containing catecholamines with 6-hydroxydopamine treatment (6-OHDA; Fig. 7b). Correspondingly, post-stroke gut permeability at 5 h was not evident in mice deficient of adrenergic receptor ADRB2 (Fig. 7c). Furthermore, goblet cell hyperplasia in the ileum of post-stroke animals was comparable to sham-operated mice at 5 h (Fig. 7d) and 24 h (Fig. 7e) following chemical sympathectomy with 6-OHDA. Additionally, the ileal crypt–villi ratio after stroke was comparable between 6-OHDA-treated sham-operated and post-stroke mice (Fig. 7f). These findings strongly suggest that signalling through the SNS promotes intestinal epithelial changes after stroke. It is noteworthy that despite the observed phenotypic changes of intestinal cells and epithelial barrier of post-stroke *Adrb2*^*-/-*^ mice and SPF mice systemically treated with 6-OHDA, there was no change in their infarct size (Supplemental Fig. 3), indicating that the lack of gut dysfunction in these post-stroke animals was not due to reduced brain damage.

**Fig. 8:**
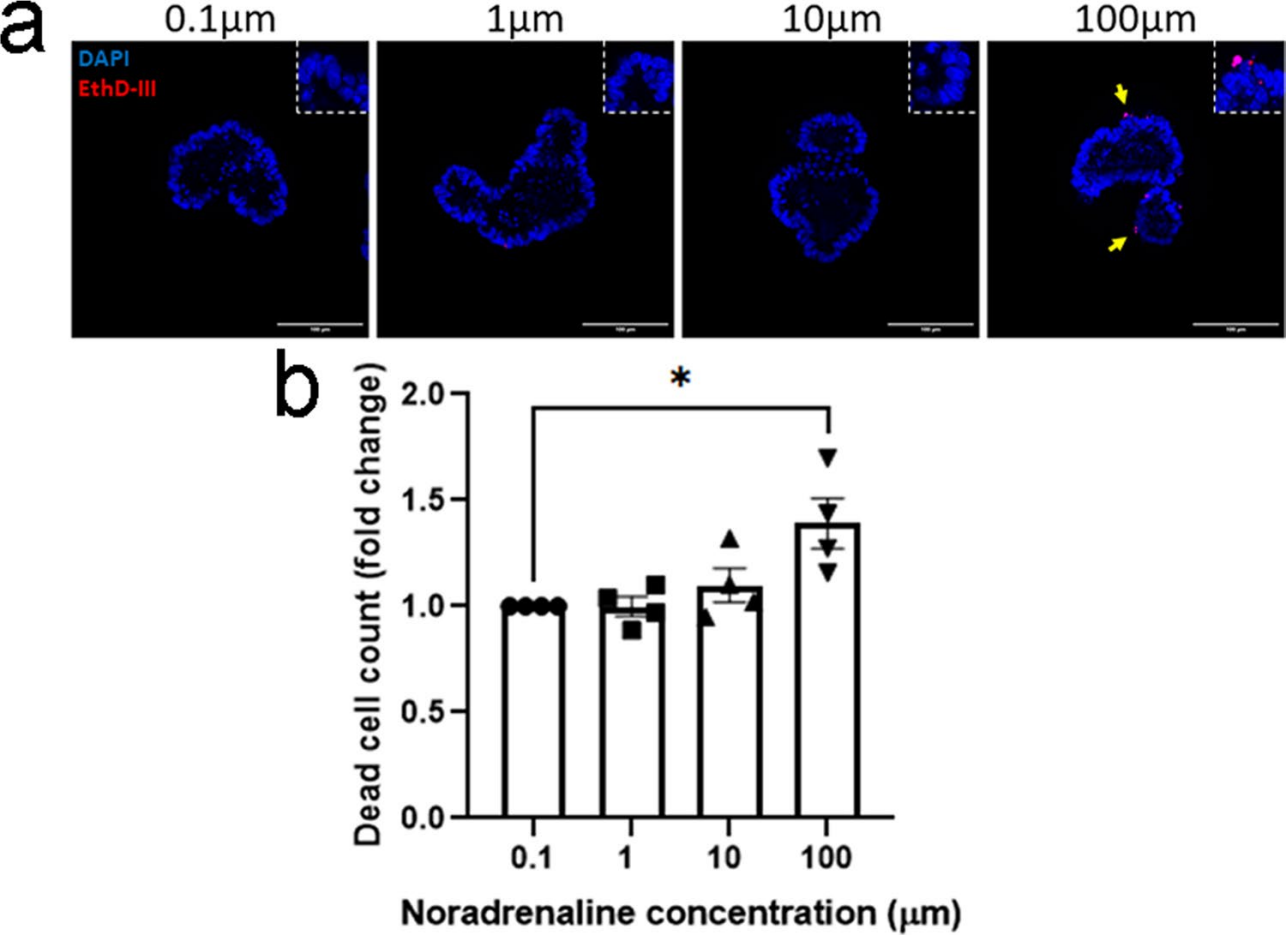
Noradrenaline induces intestinal epithelial cell death. Epithelial crypts were isolated, resuspended in Matrigel, and grown in a small intestinal culture medium. Established organoids were passaged, treated with noradrenaline (NA) at 0.1, 1, 10, or 100 μM for 5 h, and stained with EarlyTox assay. **a** Representative images of established intestinal organoids treated with various concentrations of NA, and stained with Ethidium Homodimer III (EthD-III) and DAPI (scale bar = 100 µm; yellow arrows denote EthD-III positive cell death). **b** Quantification of cell death for each treatment was performed in triplicate per experiment, and organoid experiments were completed with four independent biological replicates. *p <0.05 Friedman test

**Fig. 9:**
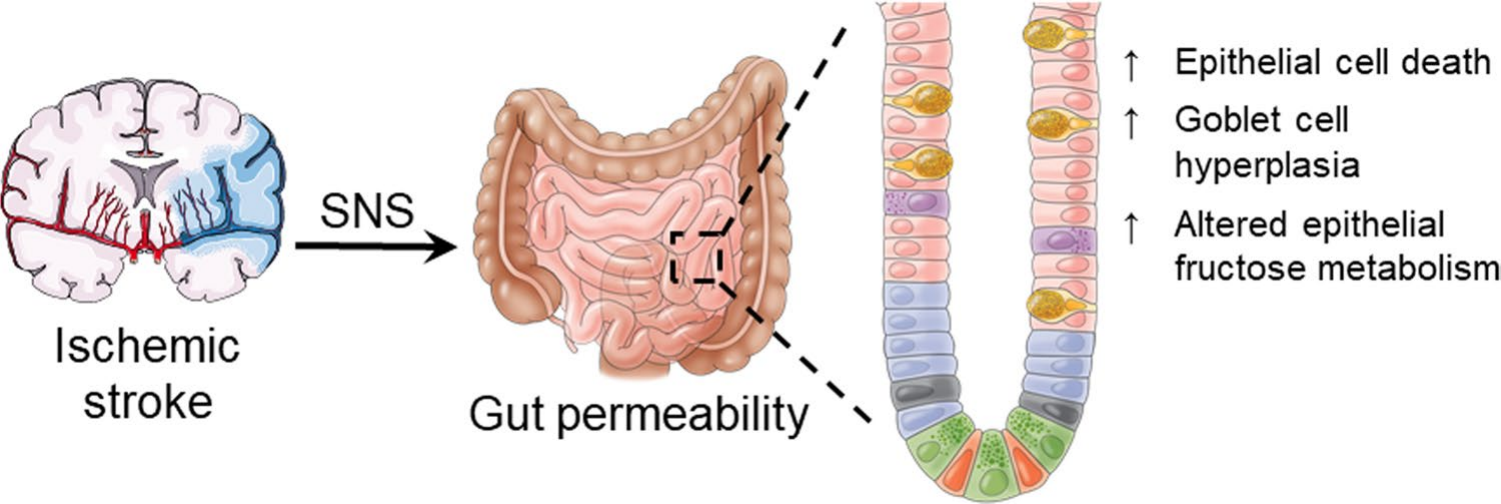
Graphical abstract summarising the major findings of the study. The mechanisms underlying gut permeability after stroke are unknown. Using an experimental mouse model, we discovered the activation of the sympathetic nervous system (SNS) contributes to stroke-induced intestinal epithelial cell death, goblet cell, and crypt hyperplasia, and altered fructose metabolism. Pharmacological denervation of sympathetic nerves returns post-stroke gut permeability to baseline levels

Sympathetic nerves in the intestines extensively innervate the enteric neurons in both the myenteric and submucosal plexi, while also innervating arterioles and lymphoid tissue, and provide sparse innervation of the mucosa directly [54]. Intestinal stem cells have been shown to express adrenoceptors [19]. In addition to *in vitro* studies, *in vivo* experiments have also suggested the activation of SNS may have a direct effect on the proliferation rate of intestinal epithelial cells [55]. To investigate whether sympathetic neurotransmitter noradrenaline could promote cell death of epithelial cells directly, we established intestinal epithelial organoids, and cultured budding organoids in the presence of NA for 5 h (Fig. 8a). It is of note that there is a basal level of NA constitutively circulating in the body [48], and as such our *in vitro* findings for epithelial cell death were expressed as fold change to 0.1 µM NA. Dead cell count within the organoids correlated with the concentration of NA treatment, with a significant increase number of dead cells in the organoids exposed to 100 µM NA (Fig. 8b). These findings suggest that direct effect of NA on intestinal epithelial cells may be responsible for the changes seen in the epithelium *in vivo* after experimental stroke. A schematic diagram summarising the major findings of the study is shown in Fig. 9.

## Discussion

Tissue injury induced by stroke is traditionally thought to be localised and limited to the brain. However, several recent seminal studies have described how stroke-mediated changes within the gut and its microbiota can prime the immune system to influence pathological outcomes (reviewed in [24]). Notably, the importance of the brain-gut axis in determining stroke outcomes was highlighted by experimental evidence that revealed gut barrier breakdown and subsequent translocation of commensal bacteria as a novel pathway for stroke-associated pneumonia [6–8]. Conceivably, understanding the mechanisms underlying stroke-induced gut barrier changes may give us an insight into their role and reveal opportunities for therapeutic targeting to limit bacterial translocation and improve clinical outcomes. In this study, we demonstrated stroke-induced gut permeability at 5 h following disease onset is attributed to robust intestinal cell death. Our findings indicated enhanced gene expression for epithelial fructose metabolism, and hyperplasia of intestinal crypts and goblet cells at 24 h after stroke, with these shifts in intestinal dynamics perhaps functioning as a host compensatory mechanism to ameliorate the impaired gut barrier integrity following brain injury. Furthermore, we discovered these effects were mediated by the activation of SNS rather than the post-stroke microbiota dysbiosis. Our study identifies a previously unknown mechanism in the brain-gut axis by which intestinal cell death and gut permeability after stroke can be rapidly counteracted by host response to restore gut barrier integrity.

Serving as the crucial interface between the luminal environment and the host, the gut barrier must be adaptable to the ever-changing intestinal environment. Indeed, localised inflammation and hypoperfusion have been identified as mediators of epithelial structure alterations associated with numerous intestinal diseases characterised by compromised gut barrier integrity [38, 39]. In this study, we did not find these factors contribute to the observed post-stroke gut permeability; however, we discovered elevated intestinal epithelial cell death, detectable with Annexin V, TUNEL, and cleaved caspase-3 staining, throughout the small intestinal tissue in the acute phase of stroke. Intriguingly, we found the presence of intestinal epithelial cell death in the ileal crypt of post-stroke animals, an observation that was almost completely absent in their sham-operated counterparts. Conceivably, these dying epithelial cells observed in normatively hyperproliferative crypts may signify an insufficient resupply of mature epithelial cells shed at the apical villus. Notably, this proposed epithelial turnover deficiency may exacerbate the structurally destabilising effects of downregulated E-cadherin expression within apical villi following stroke, further increasing the epithelial barriers vulnerability to bacterial translocation. Under homeostatic conditions, the small intestine undergoes continuous cell turnover with intestinal stem cells located in the basal crypts regularly proliferating and differentiating into specialised epithelial cells to sufficiently replenish senescent or dying cells at the villus tip [56, 57]. Accordingly, deviation from this tightly regulated epithelial cell turnover dynamic poses the risk of disrupting barrier continuity and functional integrity. Indeed, cell loss or shedding from the villus which exceeds the regenerative capacity of the crypts has been described in many pathological intestinal conditions [15]. Furthermore, studying this phenomenon is very challenging. Less than 6% of haematoxylin and eosin (H&E)–stained human small intestine sections demonstrate shedding cells in the villus [58]. Our discovery of dying epithelial cells in the ileal villus and crypt at 5 h after stroke in mice, to the best of our knowledge, is the first of its kind. Moreover, this post-stroke gut phenotype reverted to baseline or sham-operated levels by 24 h following stroke, a recovery possibly attributable to the host dynamic and adaptive response to the stroke-associated intestinal microenvironments. This robust phenotype is probably the reason why a recent study failed to detect an effect of stroke on the gut at later timepoints [59].

The major specialised intestinal epithelial subtypes, including mucus-producing goblet cells, anti-microbial producing Paneth cells, and secretory enteroendocrine cells, originate from stem cells located at the base of intestinal crypts and are derived from a common secretory precursor cell [60]. Maintaining stem cells and regulating their differentiation into distinct intestinal cell lineages involve a variety of complex signalling pathways, such as Wnt/β-catenin, Notch, PI3-kinase/Akt, and bone morphogenetic protein (BMP) signalling [61]. The findings of elevated gene expression of *Bmi1*, *Olfm4*, and *Hes1* at 5 h, and *Lgr5* at 24 h post-stroke support the observation of an increased stem cell pool and crypt size following distal brain injury. Additionally, the striking hyperplasia of goblet cells post-stroke is perhaps a feature of compensatory growth of the intestinal tract, as seen previously in surgical shortening [62]. Furthermore, the rapid differentiation and expansion of goblet cells within 5 h following stroke onset may explain the lowered expression of *Ephb2* or transit amplifying progenitor cells. Moreover, reduced gene and protein expression of Lzp in the post-stroke ileum may be part of the systemic immunosuppressive response after stroke, which has been reported to involve the activation of the sympathetic nervous system.

Indeed, additional factors, such as mucosal immune cells and cytokines [63, 64], and diet and gut microbiota [65, 66], also influence goblet cell differentiation; our findings demonstrate post-stroke goblet cell hyperplasia to be dependent on sympathetic signaling and not on microbiota changes. However, it remains to be defined how the autonomic nervous system controls intestinal epithelial cell survival, proliferation, and differentiation during the acute onset of distal injuries such as stroke. Our findings from the organoid experiments suggest that NA elicit direct effect on intestinal epithelial cells. It is possible that the effects of the sympathetic nerves on the intestinal epithelium may be mediated via submucosal neurons innervating the epithelium, such as those releasing vasoactive intestinal peptide (VIP) or acetylcholine. VIP neurons innervate the intestinal epithelium and can regulate the number of goblet cells [67]. Additionally, emerging research indicates immune cells also express adrenoceptors and respond to sympathetic innervation. Mucosal immune cells release various cytokines such as IL-22 that can regulate intestinal epithelial proliferation and barrier protection [68]. Thus, some of the impact of the sympathetic nerve activation on the intestinal epithelium may be mediated by the immune system. Further insights into the effect of stroke on goblet cell and crypt hyperplasia may reveal novel mechanisms or therapeutic targets to stimulate mucus secretion and villus lengthening to improve gut barrier integrity. Our study uncovered stroke modulates small intestinal enterocyte gene expression important for cell proliferation/differentiation and metabolism. Specifically, stroke downregulated a number of genes involved in epithelial cell proliferation and differentiation, and perhaps as a host adaptive mechanism, the predicted biological pathway enriched in these post-stroke enterocytes is associated with fructose metabolism. Whilst excess consumption of processed sugars, such as high-fructose corn syrup, is strongly implicated in diet-induced obesity [69], elevated fructose metabolism in the post-stroke enterocyte is likely to positively enhance the ability of the intestine to expand its surface, absorb nutrients, and promote cell survival [45]. In light of this, a possible therapeutic to combat gut barrier impairment after stroke is to stimulate the epithelial fructose metabolism pathway but this may not be clinically achievable considering the robustness of the stroke-induced intestinal cell death.

Despite our novel findings, there are a number of limitations in this study. The questions remained unanswered and will be the focus of future studies include: How does sympathetic innervation impact epithelial cell death and turnover in a clinical stroke setting? Is post-stroke intestinal cell death mediated through the apoptotic extrinsic pathway initiated by death receptors or via the intrinsic pathway that occurs through the mitochondria? What is the trigger for the host adaptive pathway to take effect for gut integrity restoration? It is currently difficult but will be important in future to obtain human samples to validate our experimental findings.

There is accumulating clinical evidence of microbial dysbiosis following stroke onset, but our findings indicate that the stroke-induced activation of the sympathetic nervous system mediates gut permeability and impairs barrier integrity. Investigating the process of intestinal epithelial cell death and the key initiating and regulatory factors will expand our understanding of the peripheral impacts of stroke. Furthermore, it would be intriguing to reveal if the gut barrier integrity of patients with stroke can be restored with therapeutics targeting epithelial cell fructose metabolism or mucus secretion to stimulate enterocyte survival and limit gut permeability. Uncovering the mechanisms of gut barrier dysfunction may allow future development of therapeutic and prophylactic strategies for the prevention of the serious sequelae of bacterial translocation in stroke.

## Supplementary Information

Below is the link to the electronic supplementary material.

Supplementary file1 (DOCX 3937 KB)

## Data Availability

RNA sequencing data has been made available through the GEO repository, accession #GSE216581.
