# Supplementary material for: Sympathetic-Mediated Intestinal Cell Death Contributes to Gut Barrier Impairment After Stroke
Source: Transl Stroke Res. 2023 Nov 30;16(2):280–98. doi: 10.1007/s12975-023-01211-y (PMC11976816; doi:10.1007/s12975-023-01211-y)
Supplement: Supplementary file 1 — Supplementary file1 (DOCX 3937 KB) [file 12975_2023_1211_MOESM1_ESM.docx]

**Sympathetic-mediated intestinal cell death contributes to gut barrier impairment after stroke**

Kathryn Prame Kumar^1,^*, Liam D. McKay^1,^*, Huynh Nguyen^1^, Jasveena Kaur^2^, Jenny L. Wilson^1^, Althea R. Suthya^1^, Sonja J. McKeown^2^, Helen E. Abud^2^ and Connie H. Y. Wong^1^

^1^Centre for Inflammatory Diseases, Department of Medicine, School of Clinical Sciences at Monash Health, Monash Medical Centre, Monash University, Clayton, VIC, 3168, Australia

^2^Department of Anatomy and Developmental Biology, Development and Stem Cells Program, Biomedical Discovery Institute, Monash University, Clayton, VIC, 3800, Australia.

*These authors contributed equally to this work.

**Address for correspondence:** Connie H. Y. Wong, PhD., Centre for Inflammatory Diseases, Department of Medicine, School of Clinical Sciences at Monash Health, Monash Medical Centre, Monash University, Clayton, VIC, 3168, Australia; e-mail: [connie.wong@monash.edu](mailto:connie.wong@monash.edu)

**Supplemental material and methods**

**Supplemental Table: 1**

**Supplemental Figures: 3**

**SUPPLEMENTAL MATERIAL AND METHODS**

*Characterisation of intestinal morphology and structure*

To investigate the effect of stroke on intestinal barrier integrity, the structure of the gut was assessed. Animals were culled and tissue from the duodenum to the colon was excised and the whole length of the intestine was measured. Next, a syringe was inserted into the luminal aspect of the duodenum and 10 mL of PBS was flushed through the tissue to expel faecal matter out through the distal colon. Intestinal sections were butterflied open to expose the lumen, swiss-roll and fixed in 10% neutral buffered formalin for 24-48 h at RT. Tissues were paraffin-embedded, sectioned into 4 μm slices, mounted onto a slide and stained with the hematoxylin and eosin (H&E). Macroscopic tissue inflammation was assessed in a blinded manner according to the parameters published previously [26]. Moreover, 3-7 different intact villi and crypts were randomly sampled and assessed in each ileum sample. Analysis was performed in a blinded manner on FIJI v1.53 and values were averaged for each animal.

The Alcian blue/periodic acid–Schiff (PAS) stain was used to visualise and quantify goblet cell numbers. Briefly, processed intestinal tissue were stained with Alcian blue for 15 min and rinsed with tap water for 2 min, followed by distilled water. Tissue sections were treated with periodic acid for 5 min and washed with distilled water. Tissues were then stained with Schiff's reagent for 10 min and washed with tap water for 5 min. Images of the tissue sections (5 x magnification) were taken on a Leica DM LB widefield fluorescent microscope using Imaris software, MC120 HD camera (Leica). The number of goblet cells in 3 randomly sampled whole crypt-villi units were assessed per animal. Additionally, the size of goblet cells was analysed following converting the image from RGB to 8-bit and the “Shanbhag” threshold. Analysis was performed in a blinded manner on FIJI v1.53 and values were averaged for each animal.

*Quantification of inflammatory mediators in the gut*

To investigate the influence of the intestinal immune system on the rearrangement of junctional proteins between epithelial cells and gut permeability after stroke, the protein concentrations of the cytokines tumour necrosis factor (TNF), interleukin (IL)-6 and IL-10 in the gut of sham-operated and post-pMCAO mice were measured via an OptEIA ELISA set (BD Bioscience) as per the manufacturer's protocol. Data acquisition was performed using the Infinite M1000 PRO microplate reader (Tecan). Briefly, a flat bottom 96-well ELISA plate was coated with capture antibody and incubated overnight at 4°C. The plate was then blocked using assay diluent for 1 h at RT. The top standard was added and serially diluted using assay diluent. One cm pieces of ileum tissue were homogenised with protease inhibitor for 15 sec and centrifuged at 960 g for 10 min. The sample supernatants at a volume of 100 μl were added to the wells in duplicates and incubated for 2 h at RT. The detector antibody and streptavidin HRP conjugate in assay diluent were added to each well and incubated for 1 h at RT. Samples were then incubated with TMB substrate for 30 min. Data acquisition was performed using an Infinite M1000 PRO microplate reader (Tecan). Absorption was read at 450 nm, and the resulting concentration was calculated from a standard curve. The detection limits for TNF, IL‑6 and IL-10 are as follows: 1.07 pg/mL, 3.8 pg/mL and 1.5 pg/mL. Values are expressed as pg of cytokine/mg of tissue.

*Isolation of ileal epithelial cells for quantitative PCR (qPCR)*

At experimental endpoints, mice were culled via anaesthesia overdose and cervical dislocation. The ileal tissue was dissected and opened longitudinally along the mesenteric border, washed with sterile PBS, and cut into 1 cm segments and suspended in 5 mM EDTA (pH 8.0). The suspended ileal tissue was then incubated for 1 h at 37°C at 110 *rpm* on a shaker, filtered using a 70 µm filter to isolate epithelial cells, and centrifuged at 15,000 *rpm* for 15 min at 4°C. The resulting cell pellet was then washed and stored at -80°C. To perform RNA extraction, isolated epithelial cell pellets were thawed, suspended in TRIzol (Invitrogen) for 5 min at RT, and 100 µl of 1-bromo-3-chloropropane per 1 mL of TRIzol was added to the suspension before the sample was vigorously shaken by hand for 15 s and left to incubate for 15 min at RT. The suspension was then centrifuged at 13,000 *rpm* for 15 min at 4°C, the resultant aqueous phase transferred to a fresh Eppendorf tube, and 500 µl of 2-propanol per mL of TRIzol was added before the solution was mixed by manual inversion and incubated at RT for 10 min. The suspension was then centrifuged at 13,000 *rpm* for 10 min at 4°C, the supernatant was discarded, the cell pellet was then suspended in 75% undenatured ethanol and centrifuged at 8,000 *rpm* for 5 min at 4°C, and the supernatant was removed before the cell pellet was resuspended in RNAse-free water.

Complementary DNA (cDNA) synthesis was performed in accordance with the protocol in the Superscript (SS) III cDNA synthesis kit (Promega). Briefly, up to 1 µg of RNA sample was added to a solution of 10X Reverse Transcriptase (RT) buffer (SSIII cDNA synthesis kit), MgCl_2_ (SSIII cDNA synthesis kit) and Promega RQ1 DNAse (SSIII cDNA synthesis kit), and the solution was incubated for 1 h at 37°C before Promega STOP solution (SSIII cDNA synthesis kit) was added to terminate the reaction. First strand cDNA synthesis was performed by adding random hexamers (SSIII cDNA synthesis kit) and 10 mM deoxynucleotide mix (SSIII cDNA synthesis kit) to the RNA samples before incubation for 5 min at 65°C..Samples were then placed on ice for 1 min, and a cDNA synthesis mix containing 10X RT buffer (SSIII cDNA synthesis kit), 25 mM mgCl_2_, 0.1 M dithiothreitol (SSIII cDNA synthesis kit), RNase OUT (SSIII cDNA synthesis kit), Superscript III RT (SSIII cDNA synthesis kit) and RNAse-free water was added to each sample before subsequent incubations for 10 min at 25°C, 50 min at 50°C, and 5 min at 85°C.

To quantify the gene expression of the isolated ileal epithelial cells, real-time quantitative polymerised chain reaction (qPCR) experiments were performed in accordance with the Power SYBR Green PCR Master Mix kit protocol (ThermoFisher) and analysed with a Quantistudio 6 Real-Time PCR system (Applied Biosystems). The list of primers used can be found in **Supplemental Table 1**.

### Collection of EpCAM^+^ cells from the small intestine

At 24 h after surgery, sham-operated and post-stroke mice were culled via anaesthesia overdose and cervical dislocation. The whole small intestine was placed in a slygard-lined dish containing fresh carbonated Krebs-Henseleit (KH) buffer solution. Approximately 1 cm of tissue at the proximal and distal ends of the small intestine were discarded. Under a dissection microscope, the remaining tissue was pinned using Minutien Pins (Fine Science Tools 26002-20), the fatty tissue surrounding the gut was removed, and the gut was cut along the mesenteric border. The tissues were maximally stretched (luminal side face down), pinned and flushed with KH buffer. The mucosal layer containing the mucosa and submucosa was carefully separated from the circular and longitudinal muscle layers, and incubated with 10% FCS in Ca^2+^ and Mg^2+^ free HBSS and 5 mM EDTA for 35 min at 37°C on a shaker at 110 rpm. Samples were then passed through a 70 µm filter to isolate epithelial cells. Epithelial cells were treated with Fc Block (2.4G2; BD Pharmingen) and stained with APC-conjugated anti-EpCAM (G8.8; ThermoFisher) in Ca^2+^ and Mg^2+^ free HBSS buffer containing 10% FCS and 1 mM EDTA for 20 min on ice. Samples were then washed and live epithelial cells (EpCAM^+^7AAD^-^) were collected directly into RLT buffer (RNeasy Micro kit; Qiagen) using a BD FACSAria Fusion Cell Sorter (BD Bioscience). Samples were homogenised by drawing through a 1mL syringe several times and stored at -80°C.

*Measurement of noradrenaline concentration in the small intestine.*

The concentration of noradrenaline in the ileum was determined using a commercial Norepinephrine ELISA kit (Abnova Corporation, Taipei, Taiwan, KA1891) as per manufacturer's protocol.

**Supplemental Table 1: Primer sequences for the genes assessed using qRT-PCR**

| **Genes** | **Forward sequence** | **Reverse sequence** |
| --- | --- | --- |
| *Proto-Oncogene, Polycomb Ring Finger (Bmi1)* | ATGCATCGAACAACCAGAATC | GTCTGGTTTTGTGAACCTGGA |
| *Olfactomedin 4 (Olfm4)* | AACATCACCCCAGGCTACAG | TGTCCACAGACCCAGTGAA |
| *Hairy and enhancer of split-1 (Hes1)* | ACACCGGACAAACCAAAGAC | ATGCCGGGAGCTATCTTTCT |
| *Leucine Rich Repear Containing G Protein-Coupled Receptor 5 (Lgr5)* | CCTTGGCCCTGAACAAAATA | ATTTCTTTCCCAGGGAGTGG |
| *Mucin 2 (Muc2)* | AGGGCTCGGAACTCCAGAAA | CCAGGGAATCGGTAGACATCG |
| *Ephrin type-B receptor 2 (Ephb2)* | AGAATGGTGCCATCTTCCAG | GCACATCCACTTCTTCAGCA |
| *Lysozyme (Lzp)* | GAGACCGAAGCACCGACTATG | CGGTTTTGACATTGTGTTCGC |

**SUPPLEMENTAL FIGURES**

a

b

c


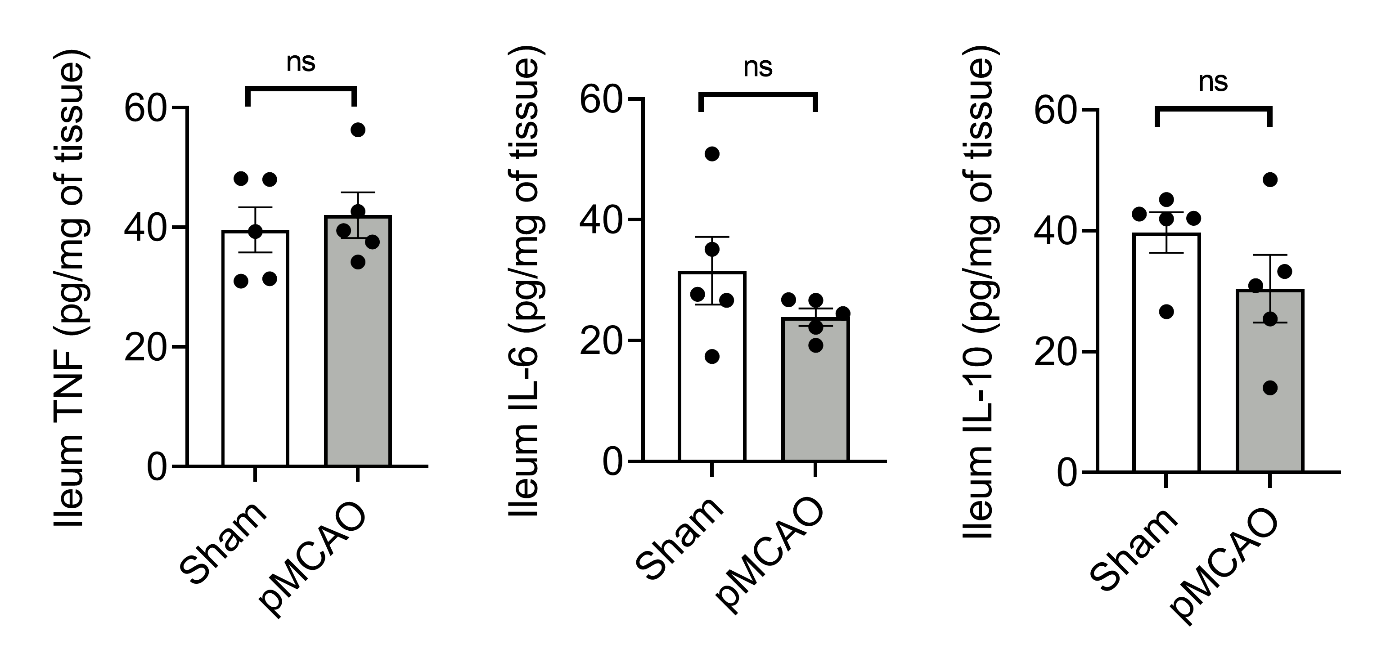


**Supplemental Figure 1: The effect of stroke on the cytokine levels in the ileum.** Mice underwent sham or permanent middle-cerebral occlusion (pMCAO) surgery, and ileum tissue (**a**) TNF, (**b**) IL-6 and (**c**) IL-10 levels were determined via ELISA at 5 h. Each data point represents the average concentration per animal. Data are shown as mean ± SEM (n = 5/group).


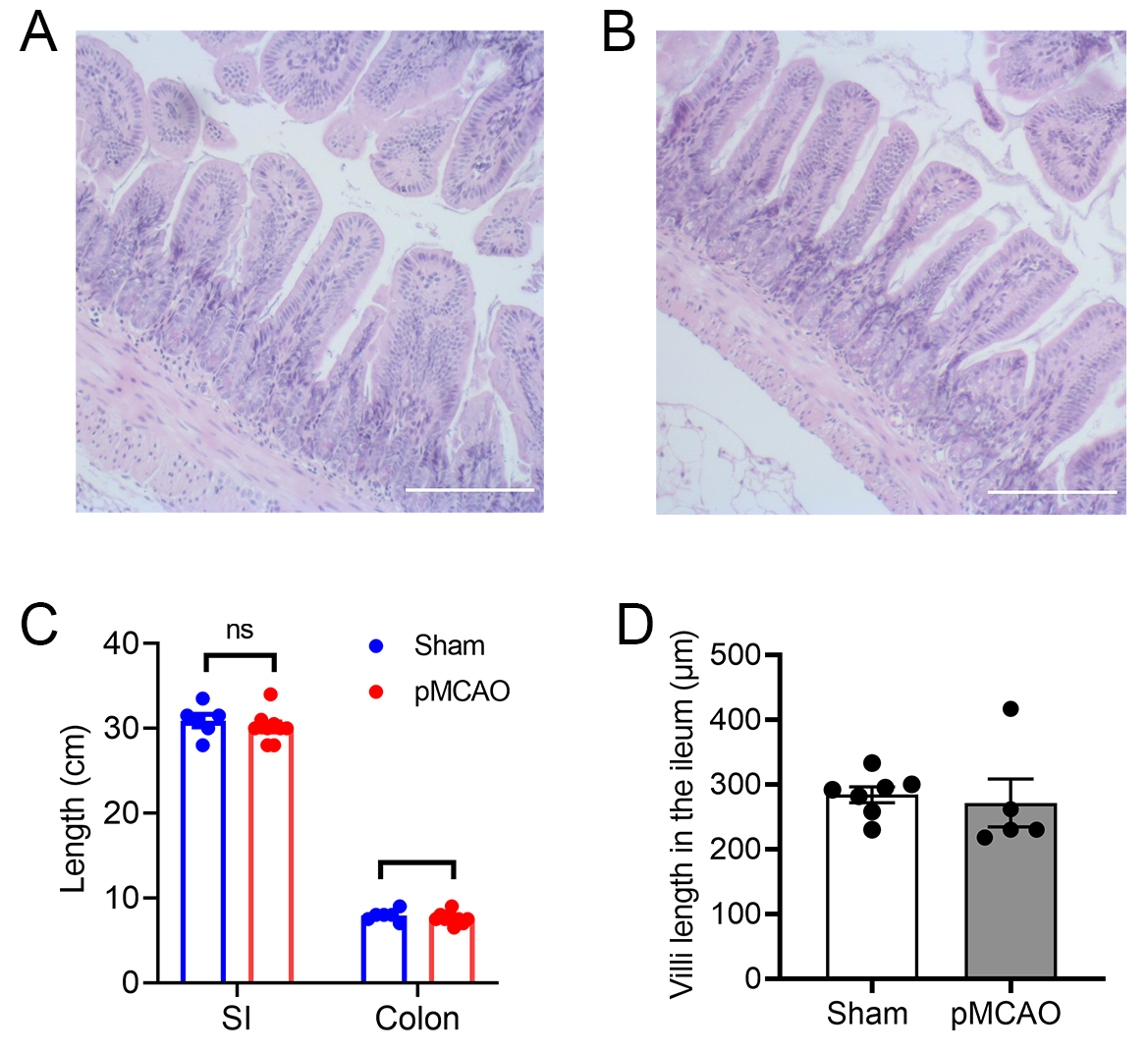


a

b

c

d

**Supplemental Figure 2: Examining macroscopic changes in the gut after stroke.** Mice underwent sham or permanent middle-cerebral occlusion (pMCAO) surgery and no histological differences were detected in the ileum at 5 h. Representative images for (**a**) sham-operated and (**b**) pMCAO ileum tissue (scale bars = 200 μm). (**c**) The small intestine (SI) and colon lengths were measured. Each data point represents the average value of one animal. Data are shown as mean ± SEM (n = 5-15). (**d**) The length of 3-6 different villi were measured per ileum sample. Each data point represents the average value of one animal. Data are shown as mean ± SEM (n = 5-16).


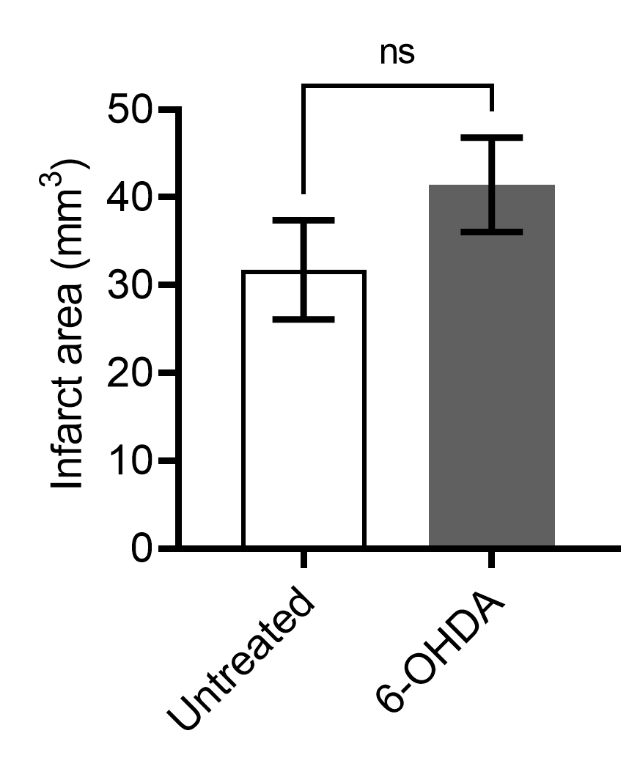


**Supplemental Figure 3: Comparable infarct size in post-stroke mice treated with or without 6‑OHDA.** Mice treated with 100 mg/kg 6-OHDA or vehicle (saline) underwent permanent middle-cerebral occlusion (pMCAO) surgery and no infarct size differences were detected in the brain at 24 h (n ≥ 14/group).
